# Chemotherapy-induced CDA expression renders resistant non-small cell lung cancer cells sensitive to 5′-deoxy-5-fluorocytidine (5′-DFCR)

**DOI:** 10.1186/s13046-021-01938-2

**Published:** 2021-04-19

**Authors:** Yanyun Gao, Philipp Zens, Min Su, Camila Anna Gemperli, Haitang Yang, Haibin Deng, Zhang Yang, Duo Xu, Sean R. R. Hall, Sabina Berezowska, Patrick Dorn, Ren-Wang Peng, Ralph Alexander Schmid, Wenxiang Wang, Thomas Michael Marti

**Affiliations:** 1 Department of General Thoracic Surgery, Inselspital, Bern University Hospital, Murtenstrasse 50, 3008 Bern, Switzerland; 2 Department of BioMedical Research, University of Bern, Bern, Switzerland; 3 Institute of Pathology, University of Bern, Bern, Switzerland; 4 Thoracic Surgery Department 2, Hunan Cancer Hospital and The Affiliated Cancer Hospital of Xiangya School of Medicine, Central South University, Changsha, 410013 Hunan China; 5 Hunan Key Laboratory of Translational Radiation Oncology, Hunan Cancer Hospital and The Affiliated Cancer Hospital of Xiangya School of Medicine, Central South University, Changsha, 410013 China; 6 Deparment of Laboratory Medicine and Pathology, Institute of Pathology, Lausanne University Hospital and University of Lausanne, Lausanne, Switzerland

**Keywords:** Chemotherapy resistant, Pemetrexed, Cisplatin, Non-small cell lung cancer, Cytidine deaminase (CDA), Thymidine phosphorylase (TYMP), 5′-DFCR, DNA damage

## Abstract

**Background:**

Pemetrexed (MTA) plus cisplatin combination therapy is considered the standard of care for patients with advanced non-small-cell lung cancer (NSCLC). However, in advanced NSCLC, the 5-year survival rate is below 10%, mainly due to resistance to therapy. We have previously shown that the fraction of mesenchymal-like, chemotherapy-resistant paraclone cells increased after MTA and cisplatin combination therapy in the NSCLC cell line A549.

Cytidine deaminase (CDA) and thymidine phosphorylase (TYMP) are key enzymes of the pyrimidine salvage pathway. 5′-deoxy-5-fluorocytidine (5′-DFCR) is a cytidine analogue (metabolite of capecitabine), which is converted by CDA and subsequently by TYMP into 5-fluorouracil, a chemotherapeutic agent frequently used to treat solid tumors. The aim of this study was to identify and exploit chemotherapy-induced metabolic adaptations to target resistant cancer cells.

**Methods:**

Cell viability and colony formation assays were used to quantify the efficacy of MTA and cisplatin treatment in combination with schedule-dependent addition of 5′-DFCR on growth and survival of A549 paraclone cells and NSCLC cell lines. CDA and TYMP protein expression were monitored by Western blot. Finally, flow cytometry was used to analyze the EMT phenotype, DNA damage response activation and cell cycle distribution over time after treatment. CDA expression was measured by immunohistochemistry in tumor tissues of patients before and after neoadjuvant chemotherapy.

**Results:**

We performed a small-scale screen of mitochondrial metabolism inhibitors, which revealed that 5′-DFCR selectively targets chemotherapy-resistant A549 paraclone cells characterized by high CDA and TYMP expression. In the cell line A549, CDA and TYMP expression was further increased by chemotherapy in a time-dependent manner, which was also observed in the KRAS-addicted NSCLC cell lines H358 and H411. The addition of 5′-DFCR on the second day after MTA and cisplatin combination therapy was the most efficient treatment to eradicate chemotherapy-resistant NSCLC cells. Moreover, recovery from treatment-induced DNA damage was delayed and accompanied by senescence induction and acquisition of a hybrid-EMT phenotype. In a subset of patient tumors, CDA expression was also increased after treatment with neoadjuvant chemotherapy.

**Conclusions:**

Chemotherapy increases CDA and TYMP expression thereby rendering resistant lung cancer cells susceptible to subsequent 5′-DFCR treatment.

**Supplementary Information:**

The online version contains supplementary material available at 10.1186/s13046-021-01938-2.

## Background

Lung cancer remains the most common cancer type and the leading cause of cancer deaths [1, 2]. Non-small cell lung cancer (NSCLC) accounts for about 85% of all lung cancers, with adenocarcinoma being the main histologic subtype accounting for approximately half of the cases [3, 4]. Chemotherapy is now recognized as an important component of treatment for all stages of NSCLC, including patients with completely resected, early-stage disease, who benefit from improved survival rates after adjuvant cisplatin-based chemotherapy [5]. The combination of Pemetrexed (MTA, commercial name ‘Alimta’) with cisplatin is recommended as gold standard therapy for adenocarcinoma lung cancer and mesothelioma patients [6] (reviewed in [7]).

MTA is a folic acid antagonist that inhibits the synthesis of the precursor purine and pyrimidine nucleotides and thus blocks DNA and RNA synthesis, thereby interfering with cancer cell proliferation [8]. Cisplatin covalently binds DNA and forms inter- and intra-strand adducts, thereby blocking DNA replication and thus cell division leading to apoptotic cell death or cellular senescence [9]. However, the occurrence of intrinsic or acquired resistance to chemotherapy is a major cause of therapeutic failure in NSCLC leading to disease progression [10]. It has been postulated that cancer stem cells (CSCs) are a subpopulation of cancer cells that feature an increased tumor initiation capacity and are frequently characterized by increased resistance to chemotherapy [11]. However, in NSCLC, we demonstrated that tumor initiation and chemotherapy resistance are features of distinct cellular subpopulations, at least within the A549 cell line [12]. In the clinical setting, the occurrence of chemotherapy resistance is a major obstacle, as non-operable tumors will invariably develop resistance that limits consecutive treatment approaches. Consequently, the identification of the molecular mechanisms underlying the development of resistance to chemotherapy offers a unique opportunity to target resistant cancer cells and thus improve therapeutic options for cancer patients.

Mitochondrial metabolism plays a pivotal role in cancer progression and chemotherapy resistance (reviewed in [13, 14]). In detail, targeting mitochondrial energetics and metabolism overcame drug resistance in acute myeloid leukemia (AML) [15, 16]. In lung cancer, high mitochondrial activity is related to chemotherapy resistance [17, 18]. Thus, aberrant mitochondrial metabolism might prove to be the Achilles heel of chemotherapy-resistant cancer cells. Indeed, antibiotics targeting mitochondria were used to eradicate CSCs, treating cancer like an infectious disease [19].

Pyrimidine nucleotides can be synthesized via the de novo or the salvage pyrimidine pathway. Depending on the growth conditions, these pathways show different activities in mammalian cells [20]. Dihydroorotate dehydrogenase (DHODH) is a key enzyme for the function of the de novo pyrimidine synthesis pathway, whose activity thus depends on the function of the mitochondrial electron transport chain (ETC) [21]. In the salvage pathway, pyrimidine nucleotides are formed by annealing preformed free bases to phosphoribosyl pyrophosphates (PRPP). For the salvage pathway, cytidine deaminase (CDA) and thymidine phosphorylase (TYMP) are two key enzymes, which catalyze the first and second step in salvaging cytidine into uridine and uracil, respectively. The cytidine analogue 5′-deoxy-5-fluorocytidine (5′-DFCR) is the activated metabolite of the oral chemotherapy drug capecitabine [22], which is used for the treatment of different types of cancer including prostate, renal, ovarian and colorectal cancer, but not for lung cancer [23]. After passage through the intestinal mucosa, capecitabine is first converted by carboxylesterase (CES) into 5′-deoxy-5-fluoro-cytidine (5′-DFCR), mainly in the liver [24]. 5′-DFCR is subsequently converted by CDA and TYMP to 5-fluorouracil (5-FU) [22, 25]. As the active form of capecitabine or 5′-DFCR, 5-FU inhibits the catalytic activity of the enzyme thymidine synthase (TYMS) thereby blocking thymidine synthesis [25]. CDA activity is crucial for capecitabine efficacy. Various polymorphisms of the *CDA* gene have been identified in the population (reviewed in [26]), which are associated with differences in capecitabine activity and efficacy [27].

In replicating cancer cells, blocked thymidine synthesis leads to nucleotide pool imbalance, which subsequently leads to DNA replication errors and activation of the DNA damage response machinery, and, if not repaired, to cell death (reviewed in [28]). Indeed, we previously showed that blocking nucleotide synthesis by MTA treatment results in the accumulation of persistent DNA damage [18, 29]. Further, we found that prolonged pretreatment with MTA enhances the anticancer efficacy of subsequent cisplatin treatment [30]. This study further revealed the existence of a chemotherapy-resistant subpopulation in the NSCLC cell line A549. In a subsequent study, we showed that the parental, non-treated A549 cell line contains phenotypically distinct subpopulations. In detail, holoclone cells are characterized by a stem-like epithelial phenotype and feature an increased tumor initiation capacity compared to paraclone cells, which feature a mesenchymal phenotype and are highly resistant to chemotherapy [12]. In addition, we found that epithelial to mesenchymal (EMT)-related plasticity exists between the A549 subpopulations. EMT is a trans-differentiation program essential for numerous developmental processes during embryogenesis enabling epithelial cells to lose cell polarity and cell–cell adhesion and to concomitantly attain mesenchymal characteristics, such as enhanced migration and invasion [31]. Consquently, EMT plays a crucial role in cancer metastasis and drug resistance [32, 33].

CDA is most commonly expressed in bone marrow and liver, and more moderately or even undetectably in other tissues [34]. Databases describe CDA as a cytoplasmic protein [35] consistent with the recycling of free pyrimidines from the cytoplasm [26]. CDA expression is epigenetically silenced in approximately 60% of cancer cell lines and tissue specimen [36]. However, treatment with 5-Aza-2′-Deoxycytidine (5-Aza-dC), a DNA methyltransferase activity inhibitor, significantly increases CDA expression in cancer cell lines derived from breast, lung ovarian and melanoma tumors with a low initial CDA expression level. In addition, restored CDA protein expression by 5-Aza-dC treatment led to a significant increase in gemcitabine resistance indicating that plasticity in nucleotide metabolism is linked with chemotherapy resistance. Indeed, it has been proposed that blocking cancer cell metabolism might increase the efficiency of chemotherapeutic agents (reviewed in [37]. However, to the best of our knowledge, it is not kown whether chemotherapy-induced plasticity of nucleotide metabolism can be exploited to overcome chemoresistance in lung cancer.

In this study, we aimed to identify an inhibitor of mitochondrial metabolism that preferentially targets chemotherapy-resistant A549 paraclone cells [12]. 5′-DFCR was found to selectively inhibit the growth of chemotherapy-resistant lung cancer cells featuring increased CDA and TYMP expression. Interestingly, we found that treatment with MTA and cisplatin, either alone or in combination, increased expression of CDA and TYMP in a schedule-dependent manner. Consequently, the schedule-dependent treatment with 5′-DFCR specifically targets resistant cancer cells that have survived the preceding MTA and cisplatin combination therapy.

## Materials and methods

### Cell culture and media

Human NSCLC cell lines A549, H358, H441, H2009, PC-9, H1993, H2228 and H3122 (CCL-185) were obtained from ATCC (American Type Culture Collection; Manassas, VA, USA) and cultured in media as recommended by ATCC (RPMI-1640 supplemented with 9% FBS, 1% Penicillin/Streptomycin solution or DMEM-F12 medium supplemented with 9% FBS, 1% Penicillin/Streptomycin and 1% L-Glutamine) at 37 °C in a humidified 5% CO_2_ incubator. A549 Rho0 cells were cultured in DMEM-F12 medium with high glucose, supplemented with 9% FBS, 1% P/S, 1% L-Glutamine, 1 mM sodium pyruvate, 50 μg/ mL uridine and 50 ng/ mL ethidium bromide. Cell lines were DNA fingerprinted and tested for mycoplasma contamination as described before [12, 30, 33].

### Cell viability assay

A549 holoclone and paraclone cells were seeded at 1000 cells per well in 96-well plates. After overnight incubation, different concentrations of the specified inhibitors of mitochondrial metabolism were added and cells were incubated for another 6 days (Table S1, Additional file 3). Cell viability was measured by using the Acid Phosphatase (APH) Assay according to the protocol described previously [38]. Changes in absorbance were recorded in a Tecan Reader Infinite M1000.

### siRNA-based gene expression silencing

siRNA-based knockdown of CDA mRNA expression (siCDA) was achieved by 3 unique 27mer siRNA duplexes (CDA Human siRNA Oligo Duplex [Locus ID 978], OriGene). Transfection of siRNAs was performed with Lipofectamine 2000 according to the manufacturer’s instructions.

### Clonogenic assay

A549 holoclone and paraclone cells, as also parental A549 cells transfected with either control siRNA (siCTRL) or siCDA, were seeded into 6-well plates at a density of 10′000 cells/well. Different concentrations of 5′-DFCR or 100 μM tetrahydrouridine (THU) were added into plates after cells attached for 6-day treatment. Subsequently, the media were removed, and cell colonies were gently washed with PBS and stained by 1% crystal violet solution (in 50% Ethanol), which was prepared from 2.3% crystal violet solution (HT901-8FOZ, Sigma). After 30 min-staining, the dye solution was pipetted back for recycling and excess dye was gently rinsed off with tap water. After the plates were air-dried, pictures were captured with a standard photo camera. Crystal violet-stained cells were dissolved in 10% acetic acid solution (2 mL/ well) for 30 min on a shaker and the concentration of the extracted dye (100 μL/ well) was quantified with a spectrophotometer at 590 nm [39].

### CDA activity assay

CDA activity assay was performed by cytidine deaminase activity assay kit (fluorometric, BioVision). Two hundred microliter assay buffer was added into 3 × 10^6^ cells. Dounce homogenizer was used to prepare the solution for activity measurement. Measurement was performed based on the protocol of CDA activity assay kit.

### Western blot analysis

NSCLC cells were seeded into 6-well plates (0.15–0.45 × 10^6^ cells per well, depending on the doubling time of the different cell lines). After 1 day (i.e. day 0), 1 μM MTA or concomitant chemotherapy treatment was used to treat cells. On day 2, 10 μM cisplatin was added for schedule chemotherapy treatment, e.g. 48 h of MTA pretreatment followed by 24 h of combined MTA and cisplatin treatment. Cells were lysed by RIPA buffer containing 1× protease and phosphatase inhibitor cocktail at different timepoints. Protein concentration was measured by Pierce™ BCA Protein Assay Kit. Equal amounts of protein lysates (11–23 μg/ lane) were resolved by SDS-PAGE. Then the bands were transferred onto nitrocellulose membranes. The membrane was firstly blocked in Intercept® (TBS) Blocking Buffer for 1 h at room temperature and then blotted with specific primary antibodies (Table S1, Additional file 3) at 4 °C overnight with shaking. Tris Buffered Saline (TBS) with 0.2% Tween-20 was used to wash the membrane. IRDye 680LT-conjugated goat anti-mouse IgG and IRDye 800CW-conjugated goat anti-rabbit IgG from Li-COR Biosciences were used at 1:5000 dilutions. Finally, signals of membrane-bound secondary antibodies were imaged using the Image Studio Lite System, also for image analysis.

### Drug response and senescence-associated β-galactosidase assay

To determine how different treatment strategies affect cell growth (Fig. 4a), 1 × 10^6^ A549 or 1.3 × 10^6^ H358 cells were seeded into 150 mm × 20 mm tissue culture-treated dishes. Starting at the day after seeding, i.e. at day 0, cells from one dish per treatment were harvested using TrypLE as negative control whereas 1 μM MTA was added to the remaining dishes. On day 2, 10 μM cisplatin was used to treat cells for 24 h. In this study, cells were treated for 3 days with 5′-DFCR at a final concentration of 200 μM. Cells were harvested by TrypLE at different time points after treatment. Cell numbers were determined using a hemocytometer and 0.1% trypan blue for dead cell exclusion. H358 cells were washed in phosphate-buffered saline and processed for analysis by flow cytometry as described below. To determine cell growth during the extended recovery period, A549 cells were harvested at day 13 of the recovery period, reseeded at a density of 0.2 × 10^6^ cells per 150 mm × 20 mm plate and cell numbers were subsequently determined as described above. Experiments for A549 were repeated independently three times. H358 cells were not harvested or reseeded, as cell numbers were still very low. Experiments for H358 cells were repeated independently two times.

Senescent cells were visualized by using the senescence β-galactosidase staining kit. In detail, 0.75 × 10^6^ chemotherapy-resistant A549 cells at recovery day 13 were reseeded in tissue culture treated 6-well dishes including treatment group untreated (1), schedule (3), schedule + 5′-DFCR D0(4) and schedule + 5′-DFCR RD2(5) (Fig. 4a). After cells were attached on the next day, A549 cells were fixed and stained according to the manufacture protocol. An inverted light microscope equipped with a 20x objective was used for visualizing and imaging senescent cells. Senescent cells were counted with the plugin ‘Cell Counter’ of the ImageJ software.

### Flow cytometry (FC) analysis

H358 cells were harvested as described above ‘Drug response assay’. An untreated control was included at every time point. A549 cells were harvested at 72 h after transfection with siCTRL or siCDA. If available, 1 × 10^6^ cells were harvested and washed with phosphate-buffered saline (PBS). First, cells were stained with antibodies against the cell surface markers, e.g. EpCAM or CD90. Then, cells were fixed with IC fixation buffer solution and permeabilized with 0.1% Triton X 100 in PBS containing 1% FBS. Prior to intracellular staining, permeabilized cells were incubated in 200 μL of PBS supplemented with 10% FBS and 0.25% Fc Receptor Binding Inhibitor Functional Grade Monoclonal Antibody for 5 min at room temperature and washed in PBS containing 1% FBS. Intracellular staining was performed with AF546-conjugated Anti-Vimentin, PerCP-eFluor 710-conjugated anti-*p*-H2AX ^Ser139^, AF-488 conjugated anti-Sox 2, or anti-CDA overnight on a rotating wheel (3 rpm) at 4 °C, protected from light. On the next day, cells were washed twice with PBS with 2% FBS and stained with secondary antibody AF-488-conjugated anti-Rabbit on a rotating wheel (3 rpm) for 30 min, to stain anti-CDA antibody. At the end, cells were wahed twice with PBS with 2% PBS and resuspended in 0.5 μg/ml DAPI in PBS containing 2% FBS to stain DNA. Geometric Mean Fluorescence intensity (GMFI) was measured on a LSR2 upgraded flow cytometer (BD Bioscience) and 10′000 events were recorded. FCS files were analyzed using FlowJo V10 (Tree Star, Inc. (Ashland, OR, USA)).

### Analysis of openly available data and public databases (TCGA), gene ontology terms analysis

The sequencing data containing the whole genome expression analysis of the three different types of A549 clones, e.g. holo-, mero-, and paraclones was previously published by our group [12]. In detail, this data is openly available as additional information to our aforementioned publication 10.1016/j.neo.2018.09.008. Kaplan Meier plot website (www.kmplot.com) was used to generate CDA and TYMP gene expression and survival curves based on survival data of lung cancer patients treated with chemotherapy. Level 3 and 4 transcriptomic and reverse-phase protein array data of cancer patients were obtained from The Cancer Genome Atlas (TCGA) cBioPortal (https://www.cbioportal.org/) and The Cancer Proteome Atlas (TCPA) [40]. A normalization step was applied to normalize between samples before downstream analysis using the methods in DESeq and Limma R packages. The gene expression and corresponding survival data were extracted for correlation and prognostic analysis using the corresponding packages in R (version 3.6.0) (‘corrplot’ and ‘Hmisc’ packages for correlation analysis; ‘maxstat’, ‘survival’ and ‘survminer’ packages for prognostic analysis). A549 holoclone and paraclone RNA- seq data was used generate volcano curve by R package, showing all mitochondrial gene (MitoCarta).

### EMT (epithelial Mesenchymal transition) and MET (Mesenchymal epithelial transition) induction in A549 and H358 cells

A549 cells (0.1 × 10^6^ / well) and H358 (0.25 × 10^6^ / well) were seeded into 6-well plates. 0.5 ng/ μL (5 days) and 0.25 ng/ μL (4 days) TGF-β were used to treat A549 and H358 cells for EMT induction, respectively. Similarly, A549 cells (0.05 × 10^6^/ well) and H358 cells (0.1 × 10^6^/ well) were seeded into 6-well plates. Ten micrometre SB431542 was used to treat A549 (3 days) and H358 (5 days) cells for MET induction. Protein was harvested after treatment, as described in part ‘Western Blot analysis’.

### Patients

Initially 118 patients with resected carcinomas of the lung were included in the study. These cases were resected at the Inselspital Bern and diagnosed at the Institute of Pathology Bern between 2000 and 2016. After exclusion of 3 cases due to missing tumor in the TMA punches, 115 patients were included for the evaluation of tumoral CDA expression. The study cohort consisted of 55 patients who received a systemic treatment prior to the resection. Due to the retrospective character of the case collection, patients therapy was heterogeneous, as previously described [41]. In addition, five cases resected and diagnosed at the Hunan Cancer Hospital were included in this study. To assure a homogeneous study cohort, only the 49/55 patients who received at least one cycle of platinum-based neoadjuvant chemotherapy were included for further analyses. The biologically matched control cohort consisted of 60 patients with carcinomas of the lung without systemic treatment prior to the resection but presence of mediastinal lymph node metastases (pN2) to guarantee a similar disease stage with the study cohort before administration of neoadjuvant therapy.

### Tissue and immunohistochemistry

A next generation Tissue Microarray (ngTMA) was constructed as previously described using formalin fixed paraffin embedded tissue [42]. Immunohistochemistry (IHC) was performed at room temperature with the fully automated staining system BOND RX® (Leica Biosystems). CDA staining was conducted using a polyclonal antibody against human CDA(Tris, 1:500) as shown in Table S1, Additional file 3.

CDA expression on tumor cells was evaluated according to Mameri et al. using H-score, the product of staining intensity and frequency of positive tumor cells [36]. Figure S14 (Additional file 1) examplifies the applied intensity scoring. Additionally, we evaluated the expression patterns of CDA in a dichotomous manner differentiating between a homogeneous and heterogeneous expression. CDA expression was assessed considering all TMA cores to account for unequal numbers of tumor cells. Only cores with at least 10 vital tumor cells were eligible for evaluation.

In 25 patients, tumor tissue pre- and post-chemotherapy was available for analysis. Whole sections of formalin-fixed paraffin-embedded adenocarcinoma tissue were used for CDA-IHC [43].

### Statistics

Statistical analyses were performed using GraphPad Prism 6.03 (GraphPad Software Inc., http://www.graphpad.com) unless otherwise indicated. In all studies, data represent biological replicates (n) and are depicted as mean values ± standard deviation (SD) or mean values ± standard error of the mean (SEM) as indicated in the figure legends. Comparison of mean values was conducted with two-tailed Student’s t test and two-way ANOVA with Tukey’s multiple comparisons test as indicated in the figure legends. In all analyses, *p* values less than 0.05 were considered statistically significant.

The Mann-Whitney-U test, Fisher’s exact test and crosstabs were used to identify significant differences of the H-Score, intensity and frequencybetween the cohorts of patient samples. Spearman correlation was performed to investigate correlation of the CDA expression with clinico-pathological parameters and Wilcoxon signed-rank test for paired samples. A two-sided asymptotic *p* ≤ 0.05 was considered significant. The statistical analysis was performed using the R software (version 4.0.2, https://cran.r-project.org).

## Results

### Chemotherapy resistant A549 paraclone cells feature increased CDA expression and are sensitive to 5′-DFCR

**Fig. 1:**
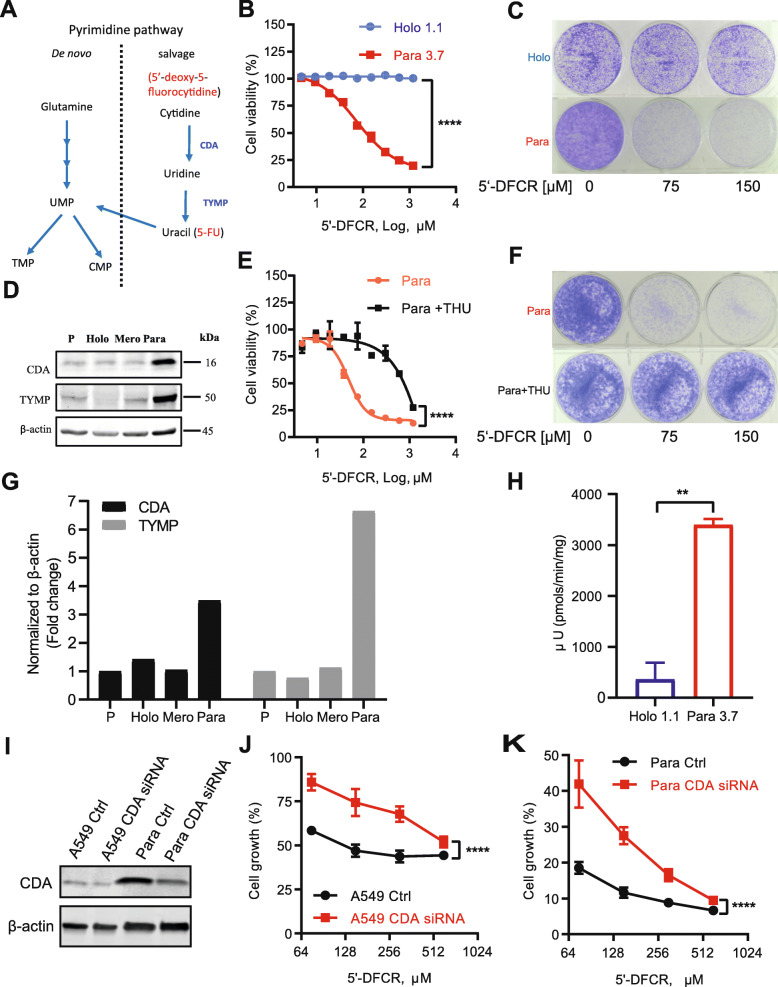
High expression of CDA and TYMP associates with chemoresistance and sensitivity to 5′-DFCR. **a** Pyrimidine pathway, CDA (cytidine deaminase) and TYMP (thymidine phosphorylase) are key enzymes in pyrimidine salvage pathway; **b** High expression of CDA and TYMP sensitized A549 paraclone cells to 5′-DFCR. Left, APH cell viability assay, right, colony formation assay; **c** CDA inhibition rescued A549 para clone cells from the sensitivity to 5′-DFCR, left APH cell viability assay; right, colony formation assay. **d** Immunoblotting of CDA and TYMP in different A549 subpopulations P: parental A549; Holo, Mero and Para represent A549 holo, mero and para clone cells, respectively. Ordinary two-way ANOVA was used for significance anlysis of drug response curves (Fig. 1**b**/**e**/**j**/**k**, ****P < .0001). A two-sided t-test was used for the significance anlysis of the CDA activity assay (Fig. 1**h**, **P < .01)

We took advantage of the morphologically and phenotypically distinct A549 subpopulations as a tool for screening mitochondrial metabolism inhibitors specifically targeting chemotherapy resistant subpopulations. Based on RNA-seq data of A549 holoclone versus paraclone cells (Figure S1D, Additional file 1), we selected six inhibitors that block various nodes of the mitochondrial metabolism (Figure S1A-C, Additional file 1), including the mitochondrial complex I inhibitor IACS-010759 (Figure S1C, Additional file 1), the pyruvate dehydrogenase (PDHα1) inhibitor CPI-613, the antibiotic tigecycline, which inhibits protein synthesis in mitochondria, the dihydroorotate dehydrogenase (DHODH) inhibitor teriflunomide, the CDA inhibitor tetrahydrouridine (THU) and the cytidine analogue 5′-deoxy-5-fluorocytidine (5′-DFCR). Of those six inhibitors, teriflunomide inhibits the de novo pyrimidine synthesis pathway, while THU and 5′-DFCR interfere with the pyrimidine salvage pathway (Figure S1B, Additional file 1). THU was selected as an inhibitor due to the increased expression of CDA in chemotherapy resistant A549 paraclone compared to holoclone cells (Figure S1D, Additional file 1). Surprisingly, only 5′-DFCR specifically targeted chemotherapy-resistant paraclone cells (Figure S2, Additional file 1). In detail, 5′-DFCR treatment significantly inhibited the proliferation and colony formation of paraclone cells, whereas holoclone cells were not affected (Fig. 1b and c, respectively). We speculated that the growth inhibitory effect of 5′-DFCR on paraclonal cells was related to the high CDA and TYMP expression levels (Fig. 1d and g) and CDA activity levels (Fig. 1h), respectively, which convert 5′-DFCR to the toxic metabolite 5-FU in two sequential reaction steps (see also Fig. 1a). Indeed, treatment with the CDA inhibitor THU was able to rescue not only short-term survival but also long-term colony formation capacity of paraclonal cells from 5′-DFCR toxicity (Fig. 1e and f, respectively). In addition, siRNA-mediated silencing of CDA expression not only reduced CDA protein levels (Fig. 1i) but also significantly rendered both parental and paraclonal A549 cells more resistant to 5′-DFCR treatment (Fig. 1i and j, respectively). In other words, the sensitivity of chemotherapy-resistant paraclonal cells to 5′-DFCR is dependent on functional CDA.

### EMT-induction increases CDA and TYMP expression

**Fig. 2:**
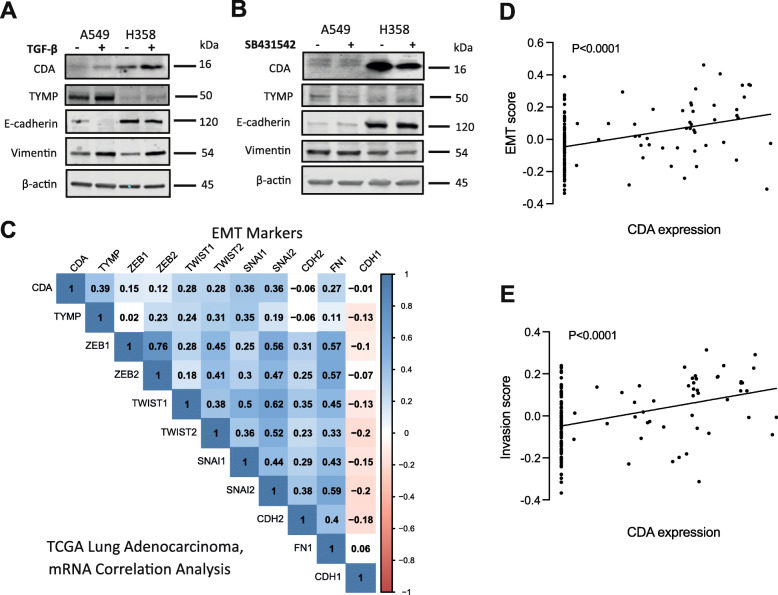
CDA and TYMP expression correlated with EMT pathway. **a** CDA and TYMP expression increased after TGF-β induced EMT transition in A549 and H358 cells, which is showed by immunoblotting (**b)** CDA and TYMP decreased after TGFβ inhibitor SB431542 induced MET transition. **c** Correlation matrix between CDA, TYMP expression and EMT transcription factors among lung adenocarcinoma from TCGA database. **d**, **e** Correlatin analysis between EMT status and CDA expression based on single cell sequencing of lung adenocarcinoma patient PDX samples. Ordinary two-way ANOVA was used for significance anlysis (*P < .05, **P < .01, ***P < .001, ****P < .0001; n.s., not significant)

Basal CDA expression levels are higher in mesenchymal paraclone compared to epithelial holoclone A549 cells (Fig. 1d and g). Silencing of CDA expression in parental A549 cells induced a phenotypic shift towards a more epithelial morphology (Figure S3E, Additional file 1), which was associated on the molecular level with an EMT-related plasticity, i.e. increased SOX2 and CD90 expression indicative of a hybrid-E/M status (Figure S2A and B, Additional file 1). We previously observed bidirectional plasticity during in vitro and in vivo cultivation of cellular A549 subtypes, e.g. holoclone cells can undergo an EMT to convert to a meroclonal and subsequently a paraclonal state whereas paraclone cells can undergo MET to convert to a meroclonal and subsequently to the holoclonal state [12]. Thus, we speculated that the CDA expression levels are positively correlated with the cellular EMT status. We took advantage of the publically available TCGA database containing 517 cases of primary lung adenocarcinoma tumors and performed an *in-silico* mRNA expression analysis of *CDA*, *TYMP* and several EMT markers (Fig. 2c). *CDA* and *TYMP* mRNA expression levels were positively correlated with mesenchymal transcription factors *TWIST2*, *SNAIL1/2*, and *FN1*, *VIM* and *AXL*, which are encoding the structural proteins fibronectin and vimentin and the AXL receptor tyrosine kinase, respectively, which are all associated with a mesenchymal phenotype [12]. Besides, the mRNA transcription levels of *CDA* and *TYMP* were negatively correlated with the epithelial transcription markers *SOX2* and *NKX2–1*, and *CDH1*, e.g. the gene encoding the epithelial marker E-cadherin (Fig. 2c). In agreement, the analysis of publicaly available single cell RNA-seq data of lung adenocarcinoma PDX revealed that CDA expression levels significantly correlated with the EMT- and invasion-score (Fig. 2d**/**e, respectively). Our extended *in-silico* analysis revealed that the increased expression of CDA and TYMP in 36 chemotherapy-treated patients was significantly correlated with a poor prognosis (Figure S4A-B, Additional file 1). Further analysis based on the overall survival of 502 lung adenocarcinoma patients, revealed that low expression of CDA correlated with a better survival, while TYMP expression levels did not correlate with survival (Figure S4C-D, Additional file 1). Thus, high basal CDA expression levels are positively correlated with a higher EMT status, e.g. a mesenchymal phenotype, and increased resistance to chemotherapy.

To further investigate the relation between CDA and TYMP expression levels and EMT induction, we used TGF-β and the TGF-β inhibitor SB431542 to induce EMT and MET, respectively (Fig. 2a-b). Treatment of A549 and H358 cells with TGF-β resulted in a more mesenchymal phenotype, e.g. E-cadherin expression decreased whereas Vimentin expression reciprocally increased (Fig. 2a). TGF-β treatment increased CDA and TYMP expression in both cell lines. After induction of MET by treatment with SB431542, Vimentin expression slightly decreased in the cell line H358, while a slight increase in E-cadherin expression was detected in the cell line A549 (Fig. 2b). After MET induction, TYMP expression slightly decreased in A549 cells whereas CDA expression significantly decreased in H358 cells. In a summary, CDA and TYMP expression positively correlate with the EMT status in lung adenocarcinoma patient samples. In addition, the induction of EMT, even in the absence of accumulation of DNA damage, can increase CDA and TYMP expression. In other words, CDA expression is increased in the mesenchymal compared to the epithelial status.

### Chemotherapy treatment increases CDA and TYMP expression and activity in lung adenocarcinoma cell lines

**Fig. 3:**
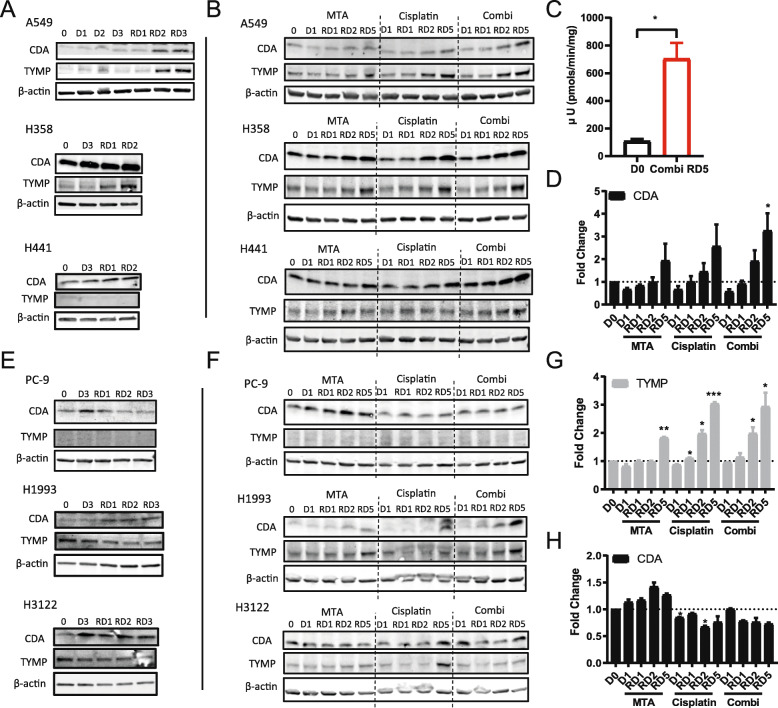
Chemotherapy treatment increases CDA and TYMP expression in a subset of lung adenocarcinoma cell lines. **a** Immunoblotting of CDA and TYMP in *KRAS* mutant cell lines A549, H358 and H441 after schedule-dependent MTA-cisplatin combination therapy (48 h MTA-pretreatment) at different time points. **b** Immunoblotting of CDA and TYMP in KRAS mutant cells after single MTA, cisplatin, and concurrent combination treatment at different time points. **c** Increased CDA enzyme activity of A549 cells after MTA and cisplatin combination treatment, two-sided student’s t test (* *p* < 0.05); **d**, **g** Quantification analysis of CDA and TYMP expression in A549 cells after single MTA, cisplatin and concomitant combination treatment compared to untreated control (see Fig. 3b), *N* = 2, two-sided student’s t test (* *p* < 0.05, **p < 0.01, ***p < 0.001). **e** Inmmunoblotting of CDA and TYMP in non-*KRAS* mutant cell lines PC-9, H1993 and H3122 after schedule-dependent MTA-cisplatin combination therapy (48 h MTA-pretreatment) at different time points; **f** Immunoblotting of CDA and TYMP in non-KRAS mutant cell lines PC-9, H1993 and H3122 after single MTA, cisplatin and concurrent combination treatment at different time points; **h** Quantification analysis of CDA expression in PC-9 after single MTA, cisplatin and concurrent combination treatment, *N* = 2, two-sided student’s t test (* *p* < 0.05)

This study revealed that CDA and TYMP protein expression is increased in chemotherapy-resistant paraclone cells compared to holoclone cells (Fig. 1d). We previously showed that treatment of the parental A549 cell line with concomitant MTA and cisplatin combination therapy increased the fraction of paraclone-like cells and that this effect could be further enhanced if MTA treatment preceedes cisplatin treatment by 48 h [30]. Therefore, we speculated that MTA and cisplatin combination therapy might increase CDA and TYMP expression, which could be exploited to target chemotherapy-resistant subpopulations by treatment with 5′-DFCR. Indeed, schedule-dependent MTA and cisplatin combination therapy increased CDA and TYMP expression in A549 cells over time, with a dramatic increase in expression levels 2 days after cessation of the drug treatment, i.e. at recovery day 2 (RD2) (Fig. 3a). CDA and TYMP expression were also increased after single MTA or cisplatin treatment, although concurrent combination therapy further augmented the effect (Fig. 3b, d, g). Interestingly, basal CDA and TYMP expression levels varied dramatically in different NSCLC cell lines including the KRAS mutant cell lines A549, H358, H441 and H2009, as well as KRAS wild type (WT) cell lines PC-9, H1993, H3122 and H2228 (Figure S5A, Additional file 1). Our extended in silico analysis that includes 189 lung cancer cell lines and patient samples revealed only a weak correlation between CDA expression levels and KRAS mutational status (Figure S5E-F, Additional file 1). Nevertheless, schedule-dependent MTA and cisplatin combination therapy increased CDA and TYMP expression over time also in the other tested KRAS mutant cell lines, e.g. H358 and H441 (Fig. 3a, Figure S6A-C, Additional file 1). However, CDA and TYMP expression did not increase after MTA and cisplatin treatment in the KRAS mutant cell line H2009 (Figure S5B-C, Additional file 1). In the tested KRAS wild-type cell lines, this chemotherapy treatment also resulted in changes of CDA and TYMP expression levels over time, but the response was more heterogeneous (Fig. 3e-f and Figure S5D, Additional file 1). In detail, CDA expression levels peaked at the end of the treatment, e.g. at day 3 (D3), in the cell lines PC-9 and H3122 whereas the CDA levels were highest during the recovery phase (RD1–3) in the cell line H1993. In summary, in cell lines featuring low basal CDA expression levels, e.g. A549, H1993 and H3122 (see Figure S5A), chemotherapy treatment clearly increased the relative CDA expression levels over time. The relative changes and the time course of CDA expression levels were more heterogeneous in cell lines featuring high basal CDA levels, e.g. H358, H441,H2009, PC9 and H2228.

Subsequently, we evaluated how the drugs individually or as concomitant combination therapy affect CDA and TYMP expression levels over time (Fig. 3b-g, and Figure S6D-E, J-K, Additional file 1). In the KRAS-mutant cell lines A549, H358 and H441 and also in the KRAS-wild type cell lines H1993 and H3122, single MTA treatment generally increased expression of CDA and TYMP during the recovery phase (RD1–5), which was also observed after single cisplatin treatment and was most pronounced after concomitant combination treatment (Fig. 3b-g). Indeed, CDA activity was significantly increased in A549 cells at recovery day 5 after combination therapy compared to untreated control (Fig. 3c). In the cell line PC-9, TYMP expression was not detectable, and CDA expression was increased by single MTA treatment whereas cisplatin or concomitant therapy did not significantly affect CDA expression levels.

In summary, chemotherapy treatment generally increased CDA and TYMP expression and activity in lung adenocarcinoma cell lines. However, CDA and TYMP expression levels did not increase after treatment in the PC-9 cell line, in which TYMP protein expression was undetectable, suggesting that the ability to augment CDA expression after treatment may depend on the baseline genetic or metabolic state of the individual cell line.

### Efficient triple combination to overcome chemotherapy resistance in lung adenocarcinoma cells

**Fig. 4:**
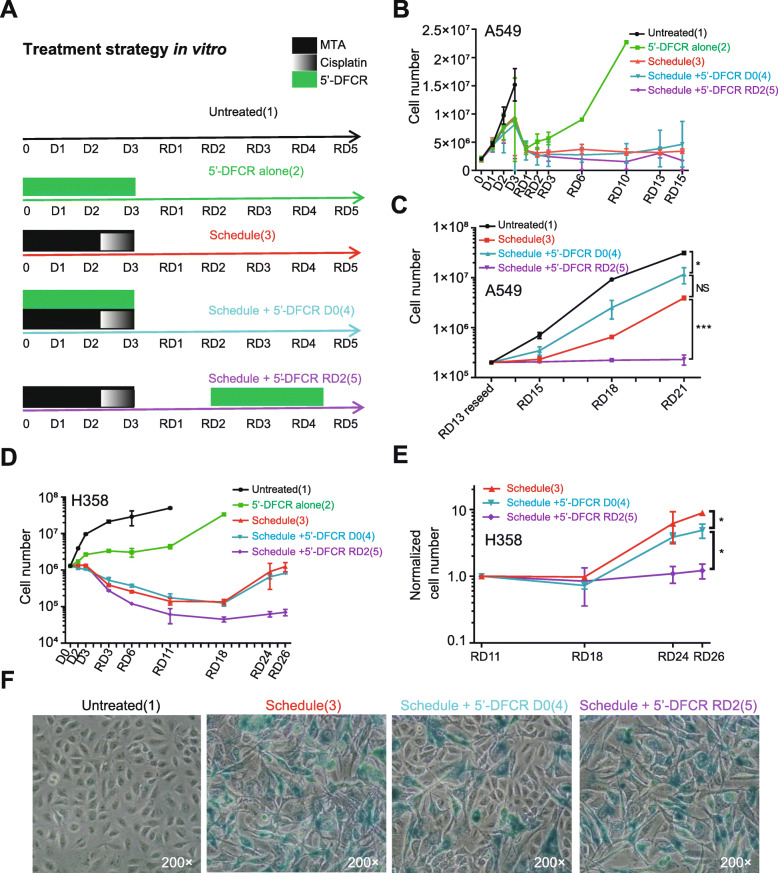
Efficient triple combination to treat *KRAS*-mutant lung adenocarcinoma cells in vitro. **a** Treatment strategies in vitro 5 treatments Untreated, 5′-DFCR alone, schedule, schedule + 5′-DFCR and schedule + 5′-DFCR were included. RD: recovery days. **b** and **c**: A549 cell growth curve after different treatments, *N* = 3, on RD21, 2-sided student’s t test was used (*p < 0.05, *** *p* < 0.001). **d** and **e**: H358 cell growth curves after different treatments, *N* = 2, 2-way ANOVA was used to compare different treatment groups (* *p* < 0.05). **f**: representative images of cells acquired by phase contrast-based microscopy at day 13 of the recovery phase (RD13) after senescence associated β-galactosidase staining (200× total magnification)

We aimed to test whether the chemotherapy-induced increase in CDA/TYMP expression (Fig. 3) can be exploited to target the remaining, chemotherapy-resistant subpopulations by subsequent treatment with 5′-DFCR (high expression of CDA conferred sensitivity to 5′-DFCR, see Fig. 1). We performed five treatment strategies including untreated (1), 5′-DFCR alone (2), schedule-dependent chemotherapy treatment either alone (3), with 5′-DFCR addition at D0 (4) or with 5′-DFCR addition at RD2 (5) (Fig. 4a). Treatment with 5′-DFCR alone did arrest cell growth only temporarily compared to control cells over time, as the A549 and H358 cells resumed their growth after drug removal (Fig. 4b and d). Treatment 3/4/5 all initially reduced the cell numbers and halted cell growth during the early recovery phase (RD1-RD15 and RD3-RD18 for A549 and H358, respectively). Pretreatment of H358 cells with chemotherapy augmented the efficiency of subsequent treatment with 5′-DFCR compared to concomitant treatment (Fig. 4d). After reseeding cells at RD13, pretreatment with chemotherapy significantly increased the efficiency of subsequent 5′-DFCR treatment compared to concomitant treatment in both A549 and H358 cell lines (Fig. 4c & e). Additionally, colony formation was completely abolished after chemo-pretreatment and subsequent 5′-DFCR treatment at RD2. In contrast, colony formation was relatively resistant to chemotherapy alone or in combination with concomitant treatment with 5′-DFCR (Figure S7, Additional file 1). In summary, treatment with chemotherapy results in increased CDA/TYMP expression during the recovery phase thereby sensitizing lung cancer cells to subsequent 5′-DFCR treatment.

### Pretreatment of 5′-DFCR treatment with chemotherapy augments senescence induction and accumulation persistence of DNA damage

We aimed to understand on the molecular level how pretreatment with chemotherapy augments the efficiency of subsequent 5′-DFCR treatment. Visual examination after treatment revealed that a significant fraction of the cells displayed morphological changes that are associated with senescence, namely increased cell size and flattened shape (reviewed in [44]). The fraction of cells, which stained positive for senescence-associated β-galactosidase activity increased after treatment with chemotherapy compared to untreated cells (Fig. 4f and Figure S7C, Additional file 1). Interestingly, the fraction of senescent cells was reduced after triple combination therapy compared to chemotherapy alone. However, the percentage of senescent cells was highest after treatment with chemotherapy followed by subsequent treatment with 5′-DFCR at RD2 (Fig. 4f and Figure S7C, Additional file 1). Detected by flow cytometry, increased forward (cell size) and side (cellular granularity) scatter intensity (F/S-high) is an additional characteristic associated with senescence (reviewed in [45]). This flow cytometric analyses revealed that the highest ratio of F/S-high versus F/S-low in H358 cells was observed after combined treatment with chemotherapy and 5′-DFCR, irrespective of the time point of 5′-DFCR addition (Figure S8H, Additional file 1). A classical hallmark of senescence is the induction of a terminal cell cycle arrest [46]. It has been shown that the increase in cell size of cancer cells after cisplatin treatment leads to shifts in the DAPI signal [30]. Therefore, we individually analyzed the cell cycle distribution of the F/S-high and F/S-low populations according to our previously established protocol ([29, 30], see also Figure S8, Additional file 1). Indeed, treatment with chemotherapy alone induced a dramatic accumulation in S-phase in both, F/S-low and F/S-high cells (Figure S9, Additional file 1). However, the level of cells in the G1-phase of the cell cycle returned to almost pretreatment levels in the F/S-low subpopulation whereas most cells of the F/S-high subpopulation remained in S- or the G2/M-phase during the extended recovery phase, e.g. RD11–26 (Figure S9, Additional file 1). Compared to treatment with chemotherapy alone, no obvious differences in the cell cycle distribution was detectable after concomitant treatment with chemotherapy and 5′-DFCR. However, after treatment with chemotherapy and subsequent 5′-DFCR treatment at RD2, the percentage of cells in the in S- or the G2/M-phase remained high during the extended recovery phase even in the F/S-low subpopulation (Figure S9, Additional file 1).

**Fig. 5:**
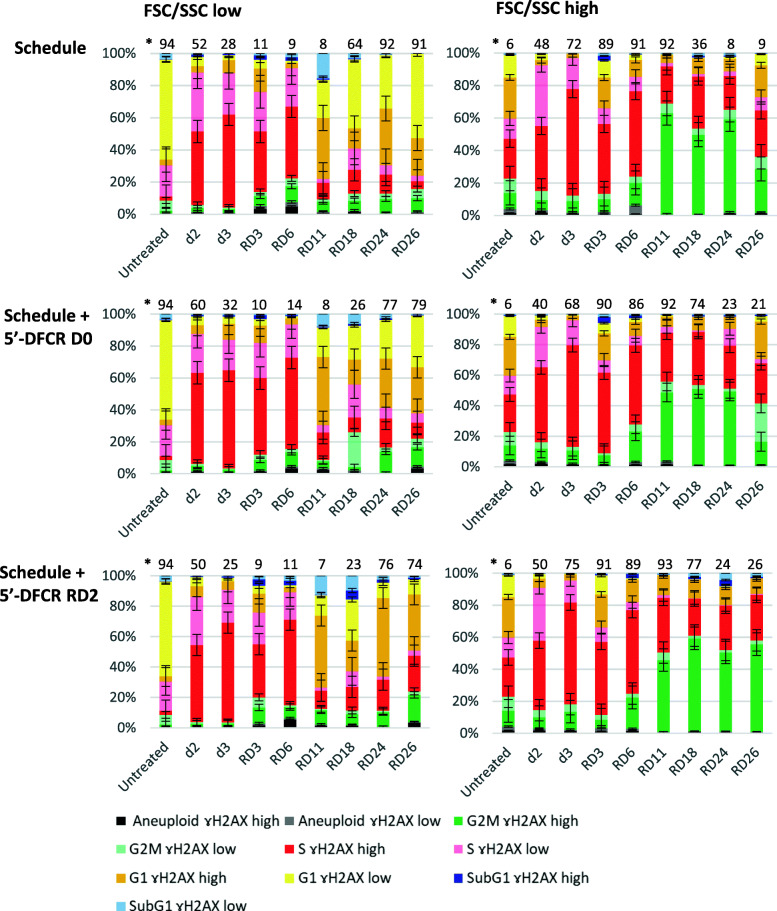
5′-DFCR at recovery day 2 after schedule-dependent treatment induced persistent DNA damage. Accumulation DNA damage within each cell cycle phase was determined in F/S-low and F/S-high cells separately. Cell cycle phases and H2AX phosphorylation levels were gated as described in supplementary Figure S8. Basal ɣH2AX was set at 10% as ɣH2AX high in untreated controls and used for normalization among experiments as described in the material and methods section. Data are represented as mean ± SD (*N* = 2)

We previously demonstrated that accumulation of persistent DNA damage leads to a cell cycle arrest and induction of senescence in lung cancer cells [29, 30, 47]. Thus, we determined the effect of the different treatment regimens on H2AX phosphorylation (ɣH2AX), a marker of DNA damage ([48], see also Figure S8, Additional file 1). As expected, treatment with chemotherapy induced H2AX phosphorylation in the majority of cells (Fig. 5, d3). During the late recovery phase (RD11–26), the level of ɣH2AX remained very high in all F/S-high cells (with the exception of cells in the G2-phase after concomitant triple therapy in which H2AX phosphorylation levels return to normal at RD26) **(**Fig. 5, right panels). In contrast, after treatment with chemotherapy alone, levels of DNA damage continuously decreased in cells of the F/S-low subpopulation during the late recovery phase (Fig. 5, left panels). After treatment with the concomitant triple therapy, ɣH2AX levels also decreased during the late recovery period in F/S-low cells, most prominently in the G1-phase (Fig. 5, left panel). However, on recovery day 26 (RD26) after combined chemotherapy and subsequent 5′-DFCR treatment, ɣH2AX was not only detectable in almost 100% of the F/S-high cells but also in roughly 90% of the F/S-low cells (Fig. 5, bottom panels). Thus, chemotherapy and subsequent 5′-DFCR treatment on RD2 resulted in the persistence of DNA damage even during the extended recovery period.

In summary, treatment with chemotherapy and subsequent 5′-DFCR treatment at RD2 leads to an increased level of persistent DNA damage associated with the induction of a senescent phenotype compared to simultaneous treatment.

### The therapy-induced increase in CDA expression is associated with a hybrid-EMT phenotype

**Fig. 6:**
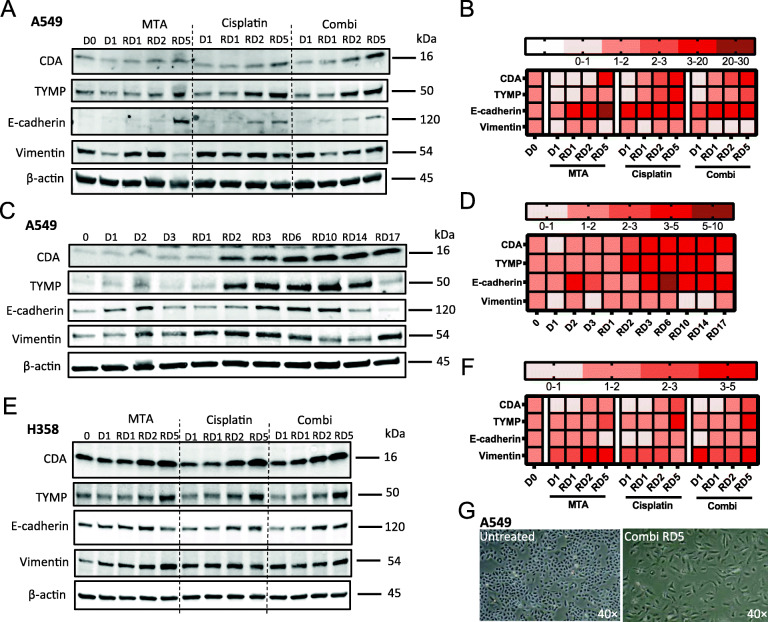
Chemotherapy induced EMT status changes along with the increase of CDA and TYMP. **a** Immunoblotting of EMT markers E-cadherin and vimentin in A549 cells after single MTA and cisplatin and concurrent combination treatment. For comparison, the corresponding immunoblotting of CDA, TYMP and β-actin are also provided (same images as in Fig. 3b, top panel). **b** Graphical categorization of the immunoblots shown in A, *N* = 3, data are represented as mean. **c** Immunoblotting of CDA, TYMP and the EMT markers E-cadherin and vimentin in A549 cells after schedule-dependent MTA and cisplatin combination treatment. **d** Graphical categorization of the immunoblots shown in C, *N* = 3, data are represented as mean. **e** Immunoblotting of EMT markers E-cadherin and vimentin in H358 cells after single MTA and cisplatin and concurrent combination treatment. For comparison, the corresponding immunoblotting of CDA, TYMP and β-actin are also provided (same images as in Fig. 3b, second panel from the top). **f** Graphical categorization of the immunoblots shown in E, *N* = 2, data are represented as mean. **g** A549 cells undergo morphological changes after MTA and cisplatin combination treatment at recovery day 5 (RD5), (40×, total magnification)

**Fig. 7:**
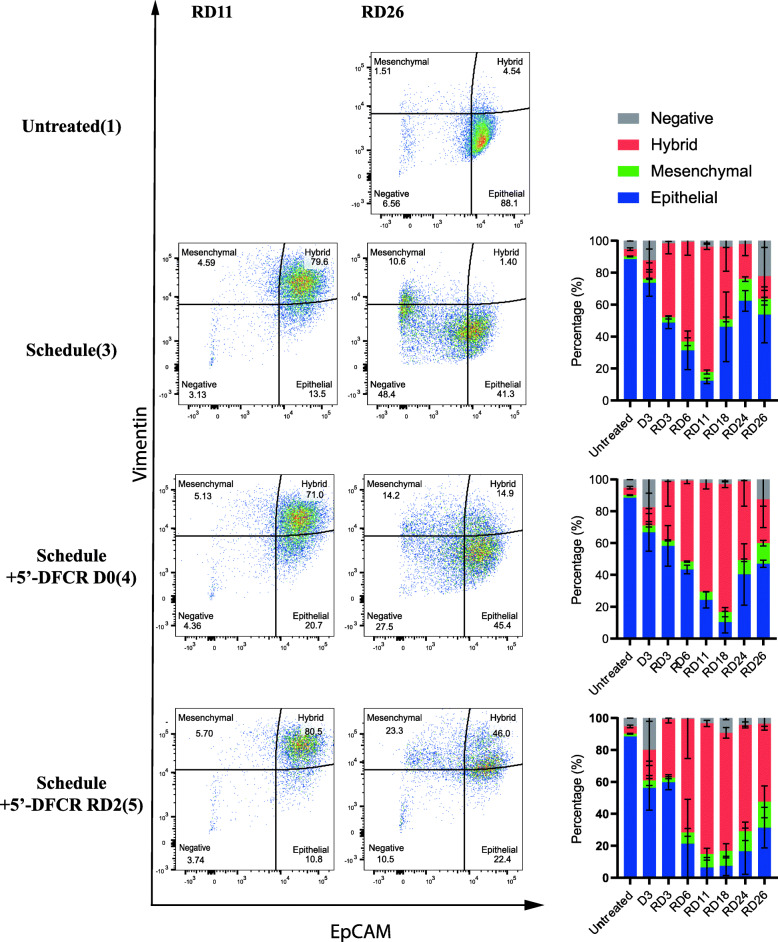
EMT phenotype plasticity of H358 cells treated with different regimens. Left panel: Flow cytometry analysis of H358 cells on recovery day 11 (RD11) and recovery day 26 (RD26) after Schedule(3), Schedule + 5′-DFCR (4), and Schedule + 5′-DFCR RD2(5) treatment. EpCAM and Vimentin were used as EMT markers to distinguish different states of EMT. Cells were featured with epithelial (EpCAM^+^/Vimentin^−^), mesenchymal (EpCAM^−^/Vimentin^+^), hybrid (EpCAM^+^/Vimentin^+^), and negative (EpCAM^−^/Vimentin^−^). Righ panel: EMT phenotype at different timepoints after treatment with different regimens. Data are represented as mean ± SD (*N* = 2)

Next, we aimed to uncover the pathways regulating the increase of CDA and TYMP expression after treatment with chemotherapy (see Fig. 2). We previously showed that treatment with chemotherapy induces an epithelial mesenchymal transition (EMT) in lung cancer cells [29, 30, 32, 43]. Thus, we hypothesized that the chemotherapy-induced activation of EMT might drive the increased in CDA and TYMP expression during the recovery phase. Expression of E-cadherin and Vimentin were previously monitored as surrogate markers to determine the epithelial and mesenchymal status of cancer cells during EMT, respectively [49]. MTA and cisplatin, either as mono- or concomitant combination treatment, increased E-cadherin expression during the early recovery phase (RD1–5) in both, A549 and H358 cells (Fig. 6a-b and e-f, respectively). Changes in Vimentin expression levels were more variable. However, combination treatment clearly induced a shift towards a more mesenchymal phenotype, which was apparent by microscopic analysis in both, the A549 and H358 cell lines (Fig. 6g & Figure S12D, Additional file 1). Indeed, a long-term time course experiment revealed that E-cadherin and Vimentin expression levels changed dynamically over time (Fig. 6c-d). Concurrent high expression levels of E-Cadherin and Vimentin indicated either the co-existence of both mesenchymal and epithelial subpopulations or the appearance of a subpopulation of cells featuring a hybrid-EMT phenotype as described by us before [12]. Indeed, our FACS analysis revealed that treatment with 5′-DFCR induced a hybrid-EMT phenotype in H358 cells (Fig. 7). After treatment with 5′-DFCR, the fraction of cells featuring a hybrid-EMT phenotype reached maximal levels at RD11 and subsequently declined during the late recovery phase (RD18–26) (Fig. 7). In contrast, the fraction of hybrid-EMT cells only reached maximal levels at RD18 after triple combination irrespective of the time point of 5′-DFCR addition. However, the disappearance of cells featuring a hybrid-EMT phenotype was significantly delayed after pretreatment with chemotherapy and subsequent 5′-DFCR treatment compared to concomitant treatment (Fig. 7, right panels).

We hypothesized that the therapy-induced hybrid-EMT phenotype might be positively associated with the high CDA expression and DNA damage levels during the late recovery phase. In agreement with our western blot results (Fig. 6e-f), our FACS analysis confirmed a time dependent increase of CDA expression and H2AX phosphorylation levels after combination therapy (Figure S11A-B, Additional file 1). The decrease of CDA-positive cells over time was delayed after triple therapy independent of the time point of 5′-DFCR treatment compared to chemotherapy alone (Figure S11A, Additional file 1). In the untreated H358 cell line, the fraction of CDA-high and ɣH2AX-high cells was higher in the hybrid subpopulation compared to the other three subpopulations (Figure S11C and D, respectively, Additional file 1). After long-term recovery (RD26) from treatment with chemotherapy and subsequent 5′-DFCR treatment at RD2, the fraction of CDA-high and ɣH2AX-high cells remained high in all except the subpopulation, which stained negative for EPCAM and VIMENTIN (Figure S11E and F, respectively, see also Figure S10I-J, Additional file 1).

In summary, all tested treatment regimens induced a hybrid-EMT phenotype that is associated with high CDA and ɣH2AX expression levels (Figure S10I-J and S11, Additional file 1). Pretreatment with chemotherapy and subsequent 5’DFCR treatment significantly enhances the persistence of this phenotype during the late recovery phase (Fig. 7 and S10I-J, Additional file 1).

### Chemotherapy elicits patient-specific changes in CDA expression in tumor tissue samples

**Fig. 8:**
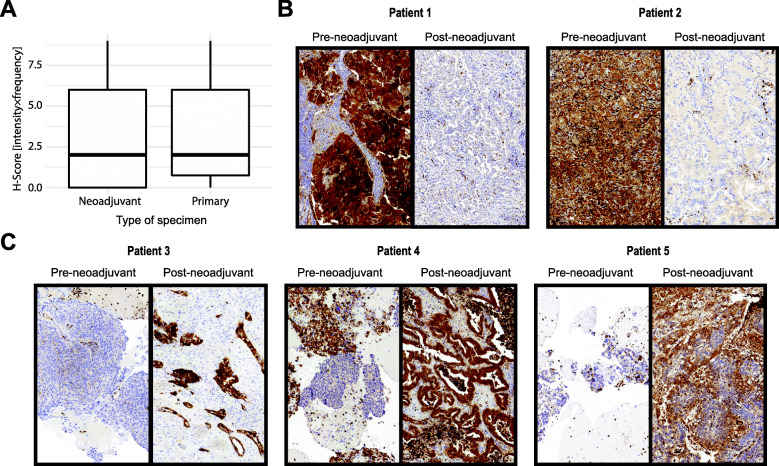
Chemotherapy exerts different effects on CDA expression in patient tumor tissues. **a**: Comparison of CDA expression between patient tumor tissue resected after neoadjuvant chemotherapy and primary resected tumors, Independent-samples Mann-Whitney U test, Wilcoxon W: 1516, Asymptotic sig. (2-sided test: 0.780); **b**: Samples with marked decrease of CDA expression after neoadjuvant chemotherapy, both preneoadjuvant specimen are lymph node biopsies. Patient 1 H-Score preneoadjuvant of 9 and postneoadjuvant of 1, Patient 2 H-Score preneoadjuvant of 9 and postneoadjuvant of 4; **c**: Samples with marked increase of CDA expression after neoadjuvant chemotherapy. Patient 1 H-Score preneoadjuvant of 1 and postneoadjuvant of 9, Patient 2 H-Score preneoadjuvant of 0 and postneoadjuvant of 9, Patient 3 H-Score preneoadjuvant of 2 and postneoadjuvant of 4

In order to evaluate the clinical significance of our finding that chemotherapy increased CDA and TYMP expression in lung cancer cells and thereby conferred sensitivity to 5′-DFCR treatment, we investigated if chemotherapy treatment also increased CDA and TYMP expression in the tumor tissues of lung cancer patients. In agreement with our western blot results (Fig. 1d), expression of CDA was lower in A549 holoclone compared paraclone cells (Figure S14E & F, respectively, Additional file 1). Next, we examined CDA expression in pre- and post-chemotherapy samples (Figure S14, Additional file 1). CDA expression of the study cohort (post-chemotherapy, mean rank = 57.49) and the control cohort (primary resected) were not significantly different (W = 1516, *p* = 0.780) (Additional file 2). There was no significantly difference between the two cohorts neither for the intensity score nor the frequency score (Fig. 8a and Figure S13, Additional files 1 and 2). CDA expression did neither correlate with pT considering all samples nor with the proportion of residual tumor cells, pN, latency between neoadjuvant chemotherapy and resection or duration of neoadjuvant chemotherapy in samples after neoadjuvant therapy. We also analyzed a paired cohort of 25 patients where matched pre- and post-chemotherapy samples of the same tumor were available. In the paired cohort, 7/25 (28%), 12/25 (48%) and 6/25 (24%) showed increased, equivalent or decreased H-scores. No significant difference was found between pre- and postneoadjuvant tissue (W = 44, *p* = 0.944). Three paired cases showed a considerable increase versus 2 cases with marked CDA decrease in the post-chemotherapy tumor tissues (Fig. 8c). The preneoadjuvant tissue of 9/25 (36%) originated from lymph nodes. Both of the two samples with marked decrease in CDA expression after neoadjuvant therapy were compared to preneoadjuvant tissue from lymph nodes suggesting a potential role of the inflammatory microenvironment on CDA expression in adenoacarcinomas of the lung.

In summary, the changes in CDA expression levels induced by chemotherapy vary significantly between cancer patients. Furthermore, CDA staining is extremely heterogeneous between different sites within a tumor and between different patients (Figure S15, Additional file 1), which led to difficulties in evaluating the effect of chemotherapy on CDA expression in patients. Nevertheless, our analysis reveals the existence of a subgroup of patients in whom CDA expression in cancer cells increases dramatically after chemotherapy.

## Discussion

### CDA expression has a bimodal distribution, which is associated with the cellular EMT status

In this study, we discovered that the chemotherapy-resistant, mesenchymal-like A549 paraclonal subpopulation features increased CDA and TYMP expression compared to the epithelial holoclonal subpopulation (Fig. 1d). Further, the A549, H1993 and the H3122 cell lines featured low but nevertheless detectable CDA epression levels whereas CDA expression levels were significantly higher in the remaining cell lines investigated in this study. Thus, we conclude that the CDA expression levels of the cell lines included in this study resemble a bimodal distribution. In agreement with these findings, our in silico analysis also revealed a binominal distribution of CDA expression in the cohort of 189 lung cancer cell lines and in primary lung cancer patient tissue samples (Figure S5E/F, respectively, Additional file 1). In agreement, an extensive in silico analysis revealed that CDA expression is downregulated in about 60% of cancer cell lines and tissues and that the low CDA expression levels correlate with CDA promoter methylation [36]. Changes in promoter methylation of genes either associated with a epithelial or a mesenchymal phenotype is a hallmark of EMT (reviewed in [50]). Indeed, we found that CDA and TYMP expression levels can be modulated by treatment with TGF-β and SB431542, which induce either an EMT or a MET, respectively (Fig. 2). Historically, it was postulated that EMT describes the bimodal transition of an epithelial to a mesenchymal state (reviewed in [50]). Thus, our findings of a bimodal distribution of the CDA expression levels in cell lines can be explained by the close association of CDA expression and the EMT status. However, our in silico analysis revealed a positive association of CDA experession with the EMT and the invasion status at the single cell, level revealing that CDA expression is very heterogenous even within the same tumor (Fig. 2d-e), which was confirmed by our study at the protein level in lung cancer patient tissue samples (Fig. 8, and Figure S14–15, Additional file 1). Further, a very recent analysis of lung cancer on the single cell level revealed eight distinct EMT-states and significant differences between EMT and MET trajectories [51]. Indeed, our analysis indicated that the therapy-induced increase in CDA expression levels is associated with the hybrid-E/M phenotype (Figure S11, Additional file 1). Further, the increase of CDA and TYMP expression after treatment with chemotherapy was more pronounced in *KRAS* mutant lung cancer cells (Fig. 3). Interestingly, KRAS mutant cancer cells that are dependent or addicted to the *KRAS* oncogene are more likely to be associated with an epithelial phenotype, while those that are independent of KRAS adopt a mesenchymal phenotype (reviewed in [52]). However, additional experiments will be required to unravel the exact molecular mechanism responsible for the positive association with CDA expression and EMT status and whether the capacity to increase CDA expression upon treatment is associated with the KRAS addition status.

### Therapy-induced stress increases CDA expression

Our study also revealed the treatment-induced increase in CDA and TYMP expression is dependent on the chemotherapeutic drug, the treatment schedule and also on the genetic and EMT-status of the individual cancer cell. In detail, CDA and TYMP expression level changes were more pronounced after cisplatin treatment compared to single MTA treatment and were further augmented by the combination therapy. Nevertheless, our in vitro experiments revealed a remarkable increase in CDA and TYMP expression during the recovery phase after treatment with chemotherapy in most of the studied cell lines. In agreement, a recent study, which analyzed the therapy-induced evolution of human lung cancer by single-cell RNA sequencing (scRNA-seq), revealed that TYMP expression was increased in progressive metastatic lung cancer compared to the NSCLC biopsy samples that were obtained from patients before initiating systemic targeted therapy [53]. The recent scRNA-seq study [53] further revealed that individual tumor and cancer cells exhibit marked therapy-induced plasticity and substantial molecular diversity. Nevertheless, the fraction of cells featuring high TYMP expression was significantly increased in on-therapy progressive disease compared to samples obtained before targeted therapy. Thus, the increased CDA and TYMP expression might not be a specific response to DNA damaging chemotherapy but might be a more general stress response associated with EMT. In agreement with this hypothesis, it has been recently reported that the CDA-dependent deoxyuridine salvage may function as anti-oxidant to protect pancreatic cancer cells against ROS [54]. Specifically, it has been suggested that uridine has an activating effect on the mitochondrial ATP-dependent potassium channel (mitoKATP) thereby reducing H_2_O_2_ levels in the mitochondria and thus the development of oxidative stress [55]. Indeed, it has been shown that CDA-dependent deoxyuridine can relieve ROS-induced endoplasmic reticulum stress to promote cancer cell survival [54]. Thus, increased CDA expression might augment cancer cell survival via the protective effect of (deoxy)uridine.

It was shown before that gene expression reprogramming during EMT is regulated mainly by epigenetic modifications [56]. Further, it has been shown that the CDA-dependent nucleotide salvage pathway displays substrate selectivity, effectively protecting newly synthesized DNA from the incorporation of epigenetically modified forms of cytosine [57]. Thus, we speculate that the deoxycytidine salvage pathway is the mechanism by which epigenetically modified nucleotides are excluded from introduction into newly synthesized DNA [58]. Active excision of epigenetically modified nucleotides by the DNA damage response machinery and subsequent repair synthesis with unmodified nucleotides will result in global DNA demethylation. Thus, we speculate that the CDA-dependent demethylation might significantly contribute to chemotherapy-induced EMT and, thereby, to the development of chemotherapy resistance. The regulation of EMT is complex, and further studies will be needed to clarify whether and, if so, how CDA is related to the chemotherapy-induced epigenetic changes underlying the development of chemotherapy-resistance.

### Exploiting therapy-induced CDA expression to specifically target resistant cancer cells

We found that treatment with chemotherapy results in increased CDA/TYMP expression during the recovery phase thereby sensitizing lung cancer cells to subsequent 5′-DFCR treatment. Interestingly, Ishitsuka and colleagues previously showed that various cytostatic drugs had increased the level of TYMP expression in WiDr human colorectal cancer xenografts, which was most pronounced by Taxol, Taxotere and Mitomycin C [59]. In this study, they also showed that Taxol/Taxotere enhanced the Capecitabine and 5’DFCR efficacy in human cancer xenografts. This group also showed that the TYMP activity and improved efficacy of Capecitabine in various human cancer xenografts can be induced by different treatment regimen including ionizing radiation [60], oxaliplatin [61], and chemoendocrine therapy [62]. Thus, our findings are in agreement with the existing literature indicating that the efficacy of Capecitabine in vivo, respectively with 5’FDCR in vitro, can be augmented by combination therapy. To the best of our knowledge, our study shows for the first time that the increased effect of the combination therapy can be further enhanced by a schedule dependent adjustment of the treatment regimen. Interesting in this context, already in 2009, a case report was published, which described a patient with metastatic lung adenocarcinoma with clear partial response to capecitabine after several lines of chemotherapy [63].

A limitation of our study is that we investigated only a limited number of possible combinations of the triple combination therapy. For example, the effect of the exact treatment schedule on the treatment efficiency has to be elucidated in more detail. It has been shown previously that the treatment schedule significantly affects treatment efficacy. In detail, the exposure of A549 cells to MTA and subsequent cisplatin treatment reduced cell survival, whereas the inversed treatment regimen or the simultaneous application of MTA and cisplatin resulted in antagonistic effects [64]. In our study, concomitant treatment with 5′-DFCR, MTA, and cisplatin resulted in a slightly antagonistic anti-cancer growth effect compared to MTA and cisplatin combination therapy alone. Only the addition of 5′-DFCR during the recovery phase, e.g., once CDA expression levels have increased, further augmented the anti-cancer effect compared to MTA and cisplatin combination therapy alone. Therefore, also in an in vivo or the clinical setting, the favorable anti-cancer effect of triple combination therapy will most likely depend on the precise treatment planning. In addition, as with any therapy consisting of multiple agents, extensive in vivo experiments will be required to determine the optimal concentration of each agent to maximize anti-cancer efficacy while limiting toxicity. Thus, we plan to perform additional experiments with cell line and patient-derived xenografts to examine the effect of the triple combination therapy in vivo. Further, it has been shown previously that the efficacy and toxicity of of Capecitabine is dependent on the enzymatic activity of Dihydropyrimidine Dehydrogenase, the rate-limiting 5-FU catabolic enzyme (encoded by DPYD, reviewed in [65]). Thus, the efficacy and toxicity of the various capecitabine-based combination therapies will consequently also depend on the genetic background of the individual tumor or patient (reviewed in [66]). Moreover, whether CDA expression increases after chemotherapy is challenging to determine in vivo since CDA expression was extremely heterogeneous in tumor tissues of patients (Figure S15, Additional file 1).

## Conclusions

In summary, we found that 5′-DFCR, the active metabolite of capecitabine, targets intrinsically chemotherapy-resistant NSCLC cells characterized by high expression of CDA and TYMP. In addition, we were able to show that treatment with 5′-DFCR also eradicates cells, which acquired resistance during the recovery phase after chemotherapy, e.g. due to increased CDA and TYMP expression. In other words, we were able to show that metabolic reprogramming after chemotherapy treatment becomes a vulnerability of resistant lung cancer cells. Altough we were not able to fully elucidate the mechanisms underlying the increase in CDA expression, we were nevertheless able to show that EMT is involved in the regulation of CDA expression. Thus, our study reveals that the chemotherapy-induced increase in pyrimidine salvage pathway expression, e.g., increased CDA and TYMP, might be exploited in the clinical setting to target therapy-resistant lung cancer by schedule-dependent treatment with Capecitabine.

## Supplementary Information

**Additional file 1: Supplementary Figure S1.** Mitochondrial metabolism inhibitors selected to target chemotherapy resistant para clone cells. A, C Inhibitors targeting different parts of mitochondrial metabolism IACS-010759 inhibiting Complex I of Electron Transport Chain, CPI-613 inhibiting pyruvate dehydrogenase ɑ1; Tigecycline, inhibiting mitochondrial ribosome 30S, blocking protein synthesis. B pyrimidine pathway related inhibitors, teriflunomide, DHODH inhibitor; tetrahydrouridine, CDA inhibitor; 5′-deoxy-5-fluorocytidine, cytidine analogue; D volcano curve based on RNA-seq data between A549 holo and para clone cells different genes related nucleotide synthesis were shown in the curve. Mitochondrial gene list (MitoCarta) was used in this curve. **Supplementary Figure S2.** Metabolic inhibitors selection to target chemotherapy resistant A549 para clone cells. Cell viability assay (APH assay), Cells were treated with different inhibitors for 6 days, Holo 1.1: A549 holoclone 1.1 cells; Para 3.7: A549 paraclone 3.7 cells. *N* = 3, two-way ANOVA was performed, * *p* < 0.05, *** *p* < 0.001, **** *p* < 0.0001. **Supplementary Figure S3**. Silencing of CDA expression increased the fraction of cells featuring a hybrid-E/M status in the A549 cell line. A Analysis of EMT-related plasticity by flow cytometry in the A549 cell line 72 h after CDA siRNA transfection, Holo-, mero-, paraclone, and hybrid cells featured a CD90−/SOX2+, CD90−/SOX2−, CD90+/SOX2−, and CD90+/Sox2+ expression phenotype, respectively; B Relative quantification for each subpopulation after CDA siRNA knockdown, *N* = 2; C Cell cycle analysis of A549 cells after siCTRL and siCDA transfection, *N* = 2; D DNA damage analysis by quantification of ɣH2AX expression levels by flow cytometry, The level of ɣH2AX expression in A549 control cells was set as 10%, *N* = 2, two sided student’s t test was used, ns: no significant difference; E Cell morphology of A549 cells 72 h after transfection with siCTRL and siCDA, respectively (40×, total magnification). **Supplementary Figure S4**. High expression of CDA and TYMP associates with poor prognosis. A and B: Kaplan-Meier Plots, correlation between CDA, TYMP expression and prognosis of patients after chemotherapy treatment; C: Kaplan-Meier univariate survival analyses of overall survival of TCGA lung adenocarnoma patients(*N* = 502), Overall survival(C and D) is analyzed and plotted using the R ‘survival’ and ‘survminer’ packages. The *p*-value is calculated by the log-rank test. **Supplementary Figure S5**. Basal expression of CDA and TYMP in different cell lines and CDA and TYMP expression after chemotherapy in additional cell lines. A Basal expression level of CDA and TYMP in different cell lines used in the study; B, C Immunoblotting of CDA and TYMP in H 2009 cells after schedule chemotherapy treatment, single treatments and concomitant chemotherapy treatment; D Immunoblotting of CDA and TYMP expression in H 2228 cells after chemotherapy treatment; E,F Comparison analysis of CDA expression in *KRAS* WT (FALSE) and *KRAS* mutated (TRUE). One hundred eighty-nine lung cancer cell lines (E) and patient samples from TCGA (F) were used in the analysis. **Supplementary figure S6**. Quantification analysis of CDA and TYMP after treatment in different cell lines (Fig. 3). A-C Quantification analysis of CDA and TYMP expression in A549, H358, and H441 cell lines after schedule treatment, *N* = 2, two-sided student’s t test, (* *p* < 0.05, **p < 0.01); D-E Quantification analysis of CDA and TYMP expression in H358 and H441 cell lines after single MTA, cisplatin and combination treatment, *N* = 2, two-sided student’s t test (* *p* < 0.05, **p < 0.01); F-H Quantification analysis of CDA and TYMP expression in PC-9, H1993, and H3122 cell lines after schedule treatment, *N* = 2, two-sided student’s t test (* *p* < 0.05, **p < 0.01); I Quantification analysis of TYMP expression in the PC-9 cell line after single MTA, cisplatin and combination treatment, *N* = 2, two-sided student’s t test (* *p* < 0.05); J and K Quantification analysis of CDA and TYMP expression in H1993 (J) and H3122 (K) cells after single MTA, cisplatin and combination treatment, *N* = 2, two-sided student’s t test (* *p* < 0.05, **p < 0.01). **Supplementary Figure S7**. Cell response comparison after three different schedule treatment strategies. A Colony morphology comparison after different treatments, A549 cell line: colony morphology and colony formation assay at recovery day 21 and 27; B Representative image of counting senescent and normal cells, ‘1’ in red represents a healthy cell, while ‘2’ in blue represents a senescent cell; C Comparison of senescent cell ratio after three different schedule treatment regimens, *N* = 3. **Supplementary Figure S8**. gating strategy for cell cycle analysis and DNA damage. A-C gating strategies for single cells; D-E: Gating strategy to distinguish small and large cells by gating FSC-SSC High and FSC-SSC low populations; F-G ɣH2AX high gate, set the gate ɣH2AX-High of untreated sample to 10% as basal level. H Comparison of FSC-SSC high population in H358 cells after different treatment strategies, *N* = 2. on RD11, two-sided student’s t test was performed to compare untreated group with the other groups, * *p* < 0.05, *** *p* < 0.001. On RD18, two-sides student’s t test was performed to compare the 5′-DFCR only group with the other three groups, * *p* < 0.05, ns: no significant difference. 2-way ANOVA was performed to compare the three groups Schedule, Schedule + 5′-DFCR D1, and Schedule + 5′-DFCR RD2, ns: no significant difference. **Supplementary Figure S9.** Cell cycle distribution of three different treatment strategies. Cell cycle distribution was analysed in F/S-low and F/S-high cells Group, respectively. ‘5’-DFCR at recovery day 2′ increased long-term S-phase and G2/M-phase arrest induced by schedule-dependent treatment**.** Data are represented as mean ± SD (*N* = 2). **Supplementary Figure S10**. Gating strategy for analyzing ɣH2AX and CDA expression levels within different EMT state. A-C: gating strategies for single cells; D: Gating for EMT state including epithelial, meshenchymal, hybrid, and negative; E, F: Under the gate ‘single cells’, CDA-High and ɣH2AX-High were gated, while roughly 10% was set for untreated H358 cells. G, H: From each status of EMT, the same gates ‘CDA-High’ (E) and ‘ɣH2AX-High’ (F) were used to evaluate CDA and ɣH2AX expression levels within different EMT state; I, H: Percentages of ‘ɣH2AX-High’ and ‘CDA-High’ in hybrid-EMT status at different time points, after different regimen treatment. High levels of ɣH2AX and CDA were shown in the hybrid-EMT status. *N* = 2, 2-way ANOVA was performed, ns: no significant difference. **Supplementary Figure S11**. Schedule+ 5′-DFCR RD2 regimen delayed the decrease of CDA and ɣH2AX expression levels. A-B: Total CDA and ɣH2AX expression over time after three regimen treatments, including schedule, schedule + 5′-DFCR D0, and schedule + 5′-DFCR RD2, *N* = 2, two-way ANOVA was performed, * *p* < 0.05, ns: no significant difference; C,D: Untreated H 358 cells at Hybrid-EMT harbored higher expression of CDA and ɣH2AX, compared with other EMT state, *N* = 2, one-way ANOVA was performed to compare mesenchymal, hybrid, and negative with the epithelial population, ** *p* < 0.01, *** *p* < 0.001; E, F: On recovery day 26 (RD26) after treatment of different regimens, CDA and ɣ H2AX levels of H 358 cells at different EMT states, *N* = 2, one way ANOVA was performed to compare mesenchymal, hybrid, and negative with the epithelial population, * *p* < 0.05. **Supplementary Figure S12**. DNA damage persistence during recovery phase after chemotherapy treatment in different cell lines A Immunoblotting of CDA, EMT markers and DNA damage marker p-H2AX expression in H 2009 cells after chemotherapy treatment; B-C Immunoblotting of CDA, TYMP and p-H2AX expression in H358 and H441 cells after chemotherapy treatment; D: morphology change H358 cells after MTA and cisplatin combination chemotherapy treatment at recovery day 5 (40×, total magnification). **Supplementary Figure S13**. No correlation of CDA expression an clinico-pathologic characteristics. A: No correlation of CDA expression and the pT stage of the tumors (109 samples included, Spearman correlation, *p* = 0.893); B: No correlation of CDA expression and the pN stage of the tumors, all primary resected samples were excluded due to a minimum pN2 (49 samples included, Spearman correlation *p* = 0.792). **Supplementary Figure S14**. Representative immunohistochemical CDA staining in patient tissues and A549 cells. A-D: Intensity scoring of CDA expression: A strong; B moderate; C weak; D negative sample; E-F Establishment of CDA staining protocol with A549 holo and para clone cells. **Supplementary Figure S15**. Heterogeneous CDA expression in patient tumor tissues. A Heterogeneity within the same TMA core, B-C: Heterogeneity in two different TMA cores of the same case; D, G: Heterogeneous CDA expression in two different cases; E, F: heterogeneity in two different TM -cores of the same case; H: Number of homogeneous and heterogeneous CDA-staining patterns in 109 patient tumor tissues (Fisher’s exact *p = 0.228)*.

**Additional file 2.**

**Additional file 3 **: **Table S1**. Key resources

## Data Availability

All data generated or analysed during this study are included in this published article and its supplementary information files.

## Abbreviations

5′-DFCR: 5′-deoxy-5-fluoro-cytidine
5-FU: 5-fluorouracil
ADC: Adenocarcinomas
AML: Acute myeloid leukemia
APH: Acid phosphatase assay
CDA: Cytidine or deoxycytidine deaminase
CES: Carboxylesterase
CSCs: Cancer stem cells
DDR: DNA damage response
DHODH: Dihydroorotate dehydrogenase
EMT: Epithelial-to-mesenchymal transition
ETC: Electron transport chain
MET: Mesenchymal to epithelial transition
MTA: Multi-targeted antifolate
NSCLC: Non-small cell lung cancer
KRAS: Kirsten RAt Sarcoma virus, oncogene
TCGA: The Cancer Genome Atlas
TGF-β: Transforming growth factor-β
THU: Tetrahydrouridine
TS: Thymidylate synthase
TYMP: Thymidine phosphorylase
γ-H2AX: Phosphorylation of histone H2A variant
