# Supplementary material for: Chemotherapy-induced CDA expression renders resistant non-small cell lung cancer cells sensitive to 5′-deoxy-5-fluorocytidine (5′-DFCR)
Source: J Exp Clin Cancer Res. 2021 Apr 19;40:138. doi: 10.1186/s13046-021-01938-2 (PMC8056724; doi:10.1186/s13046-021-01938-2)
Supplement: Supplementary file 1 — Additional file 1: Supplementary Figure S1. Mitochondrial metabolism inhibitors selected to target chemotherapy resistant para clone cells. A, C Inhibitors targeting different parts of mitochondrial metabolism IACS-010759 inhibiting Complex I of Electron Transport Chain, CPI-613 inhibiting pyruvate dehydrogenase ɑ1; Tigecycline, inhibiting mitochondrial ribosome 30S, blocking protein synthesis. B pyrimidine pathway related inhibitors, teriflunomide, DHODH inhibitor; tetrahydrouridine, CDA inhibitor; 5′-deoxy-5-fluorocytidine, cytidine analogue; D volcano curve based on RNA-seq data between A549 holo and para clone cells different genes related nucleotide synthesis were shown in the curve. Mitochondrial gene list (MitoCarta) was used in this curve. Supplementary Figure S2. Metabolic inhibitors selection to target chemotherapy resistant A549 para clone cells. Cell viability assay (APH assay), Cells were treated with different inhibitors for 6 days, Holo 1.1: A549 holoclone 1.1 cells; Para 3.7: A549 paraclone 3.7 cells. N = 3, two-way ANOVA was performed, * p < 0.05, *** p < 0.001, **** p < 0.0001. Supplementary Figure S3. Silencing of CDA expression increased the fraction of cells featuring a hybrid-E/M status in the A549 cell line. A Analysis of EMT-related plasticity by flow cytometry in the A549 cell line 72 h after CDA siRNA transfection, Holo-, mero-, paraclone, and hybrid cells featured a CD90−/SOX2+, CD90−/SOX2−, CD90+/SOX2−, and CD90+/Sox2+ expression phenotype, respectively; B Relative quantification for each subpopulation after CDA siRNA knockdown, N = 2; C Cell cycle analysis of A549 cells after siCTRL and siCDA transfection, N = 2; D DNA damage analysis by quantification of ɣH2AX expression levels by flow cytometry, The level of ɣH2AX expression in A549 control cells was set as 10%, N = 2, two sided student’s t test was used, ns: no significant difference; E Cell morphology of A549 cells 72 h after transfection with siCTRL and siCDA, respectively (40×, tot [file 13046_2021_1938_MOESM1_ESM.pdf]

Supplementary Figure 1

A

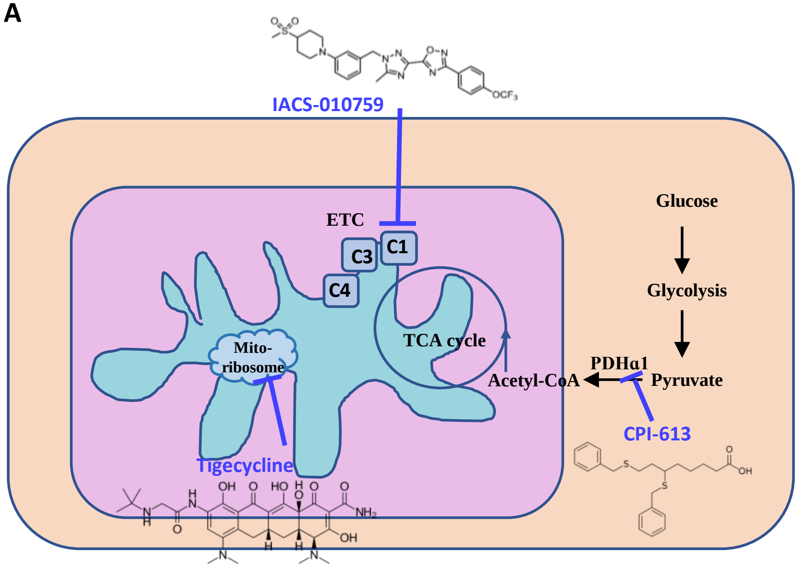

B

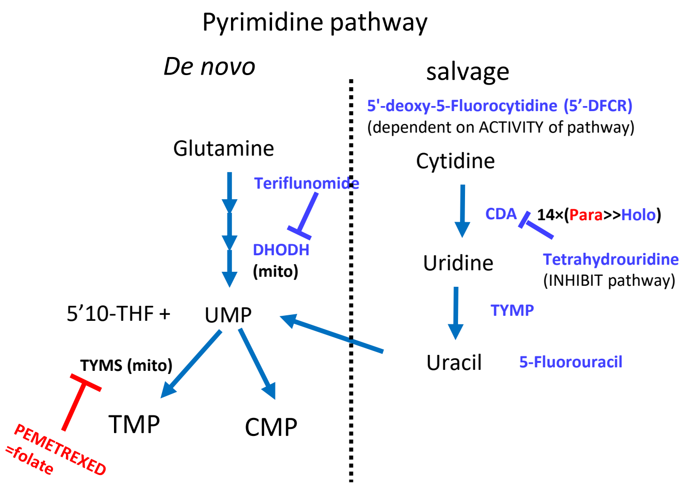

C

Number	Inhibitors	Targets
1	IACS-010759	Complex I of Electron Transport Chain (ETC)
2	CPI-613	PDHα1 (pyruvate dehydrogenase α) Pyruvate → Acetyl-CoA
3	Tigecycline	Mitochondrial Ribosome 30S
4	Teriflunomide	dihydroorotate dehydrogenase(DHODH)
5	Tetrahydrouridine	Inhibit cytidine deaminase (CDA)
6	5'-deoxy-5-Fluorocytidine	Rely on the enzymes CDA and TYMP(thymidine phosphorylase) to produce 5-Fluoro-Uracil, 5-fluoroUMP, which is a toxic analog of UMP (substrate of the mitochondrial protein TYMS)

D

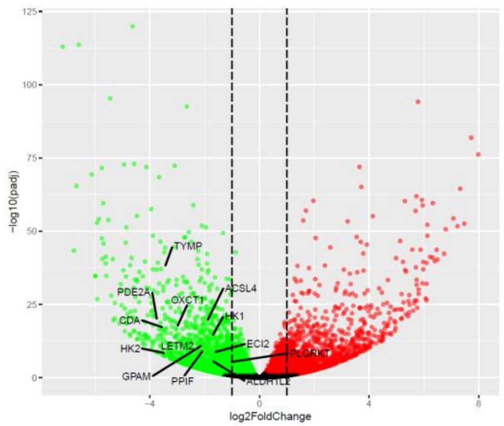

Supplementary Figure 2

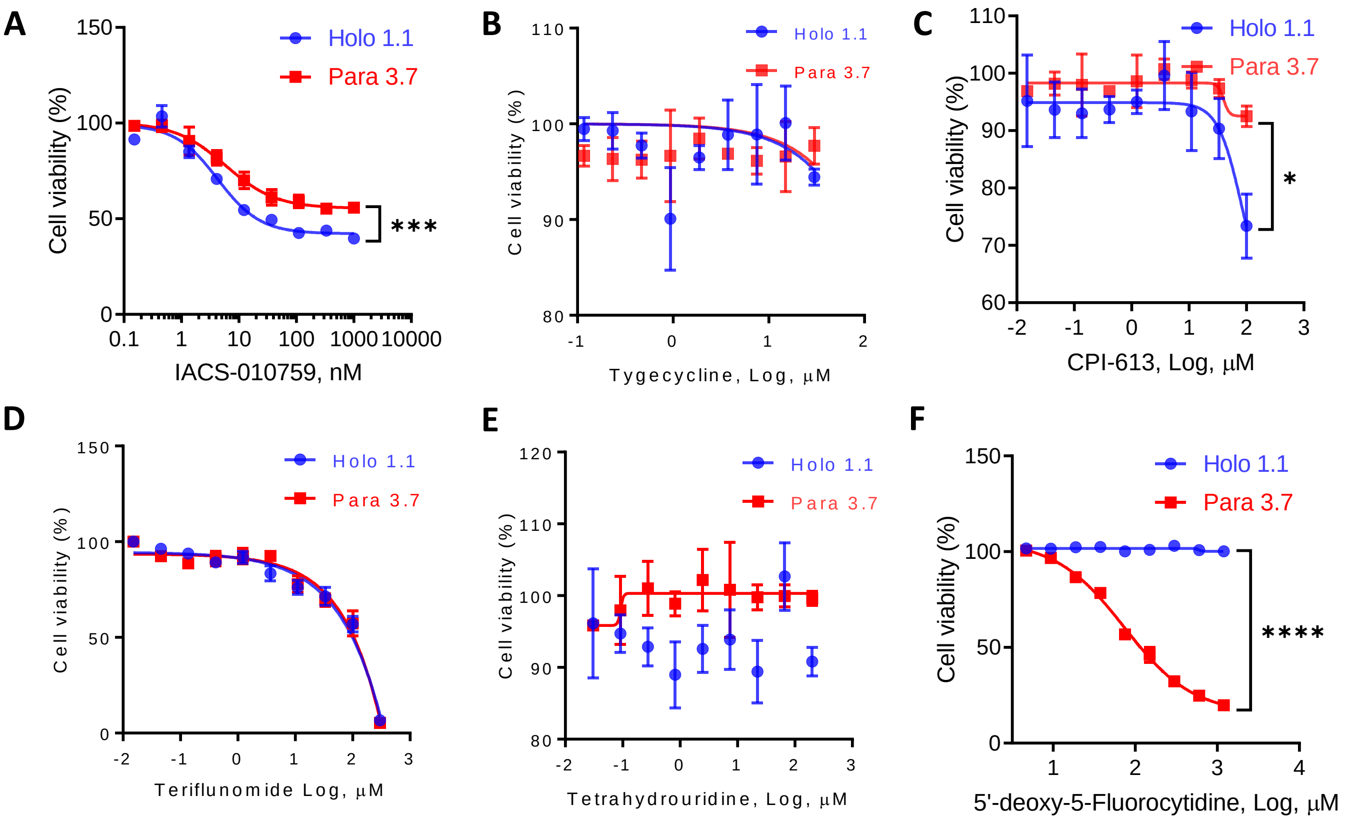

Supplementary Figure 3

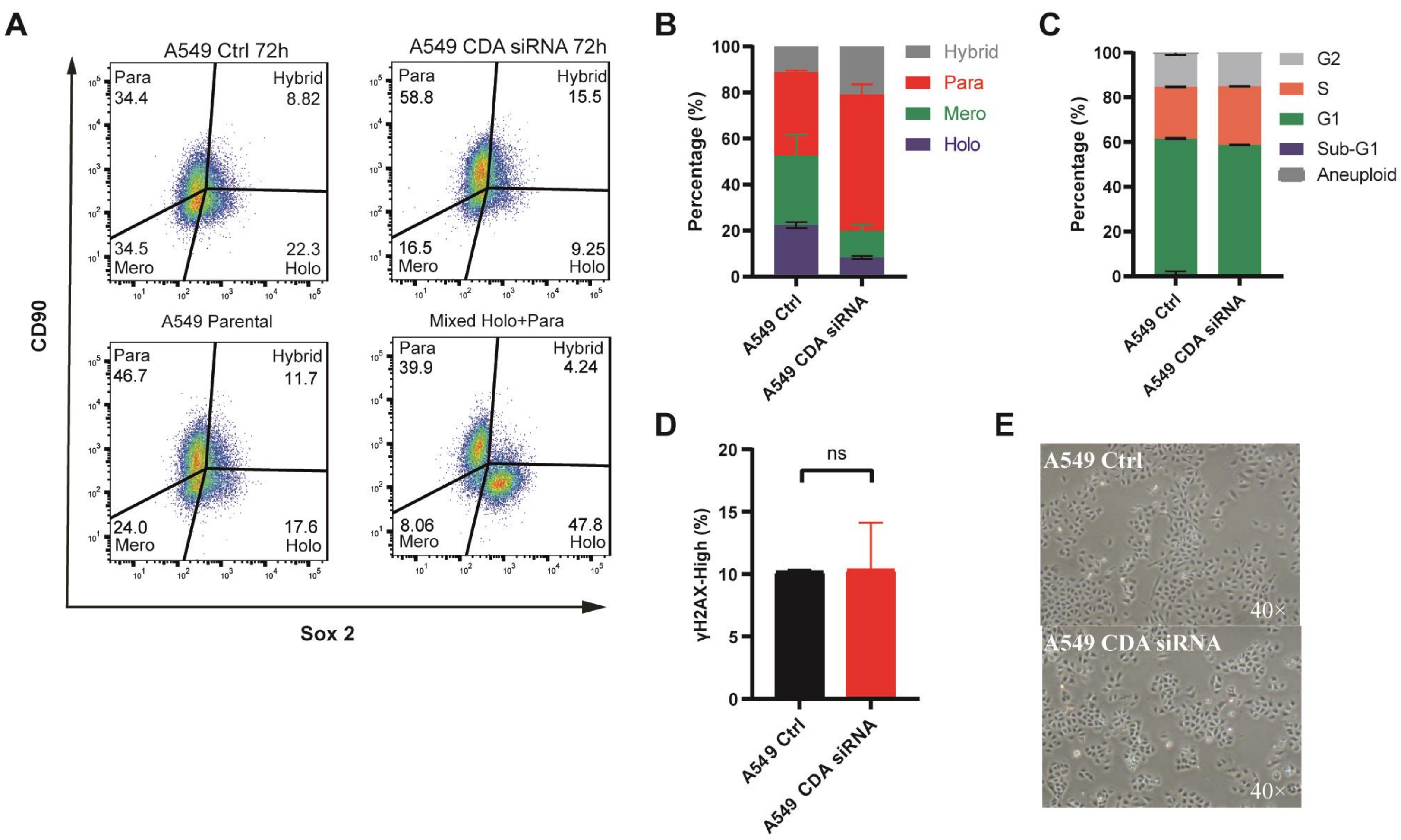

Supplementary Figure 4

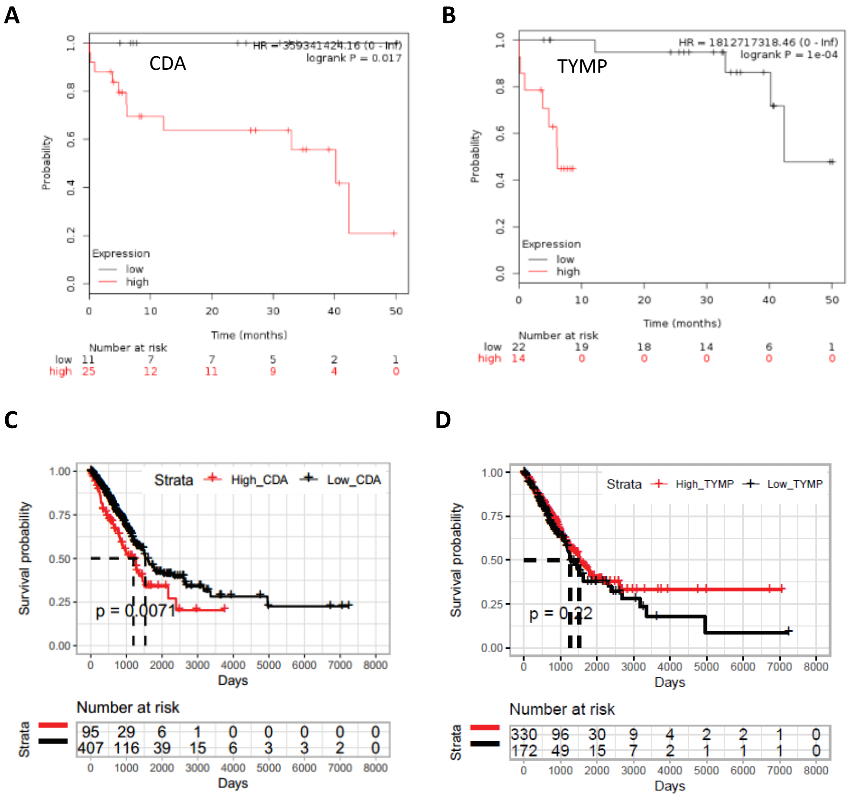

Supplementary Figure 5

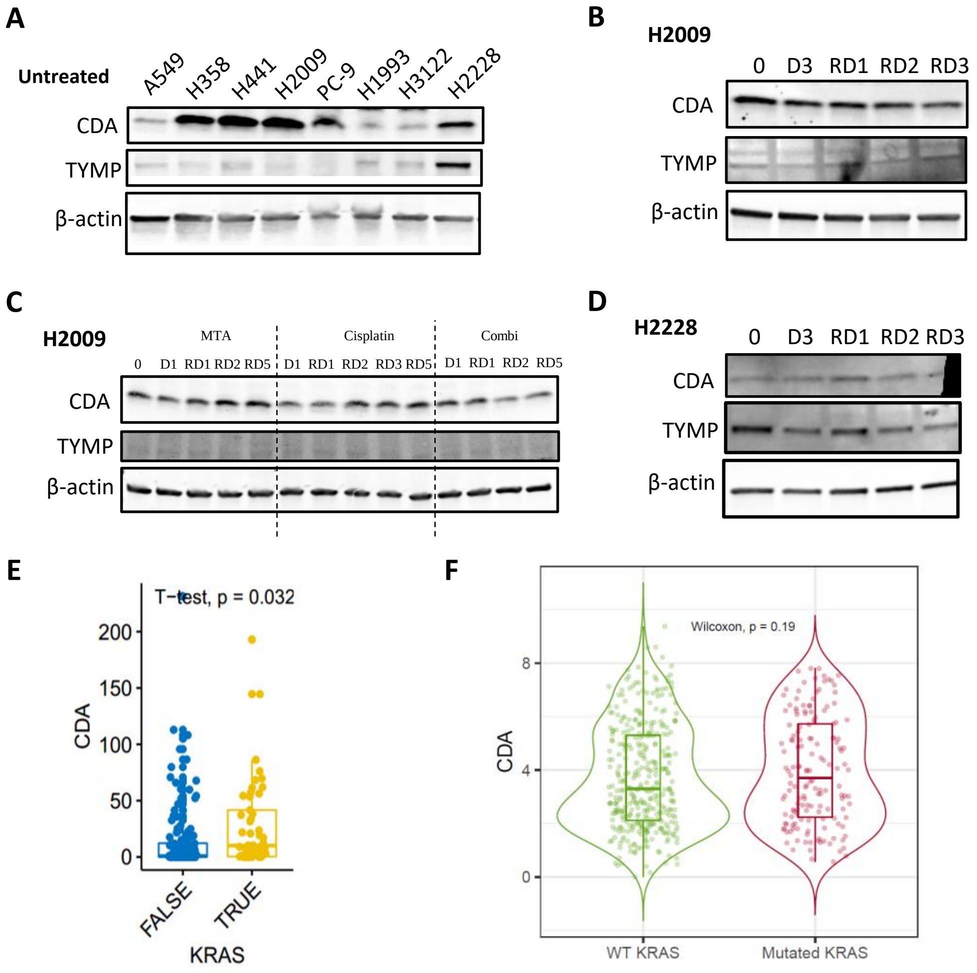

Supplementary Figure 6

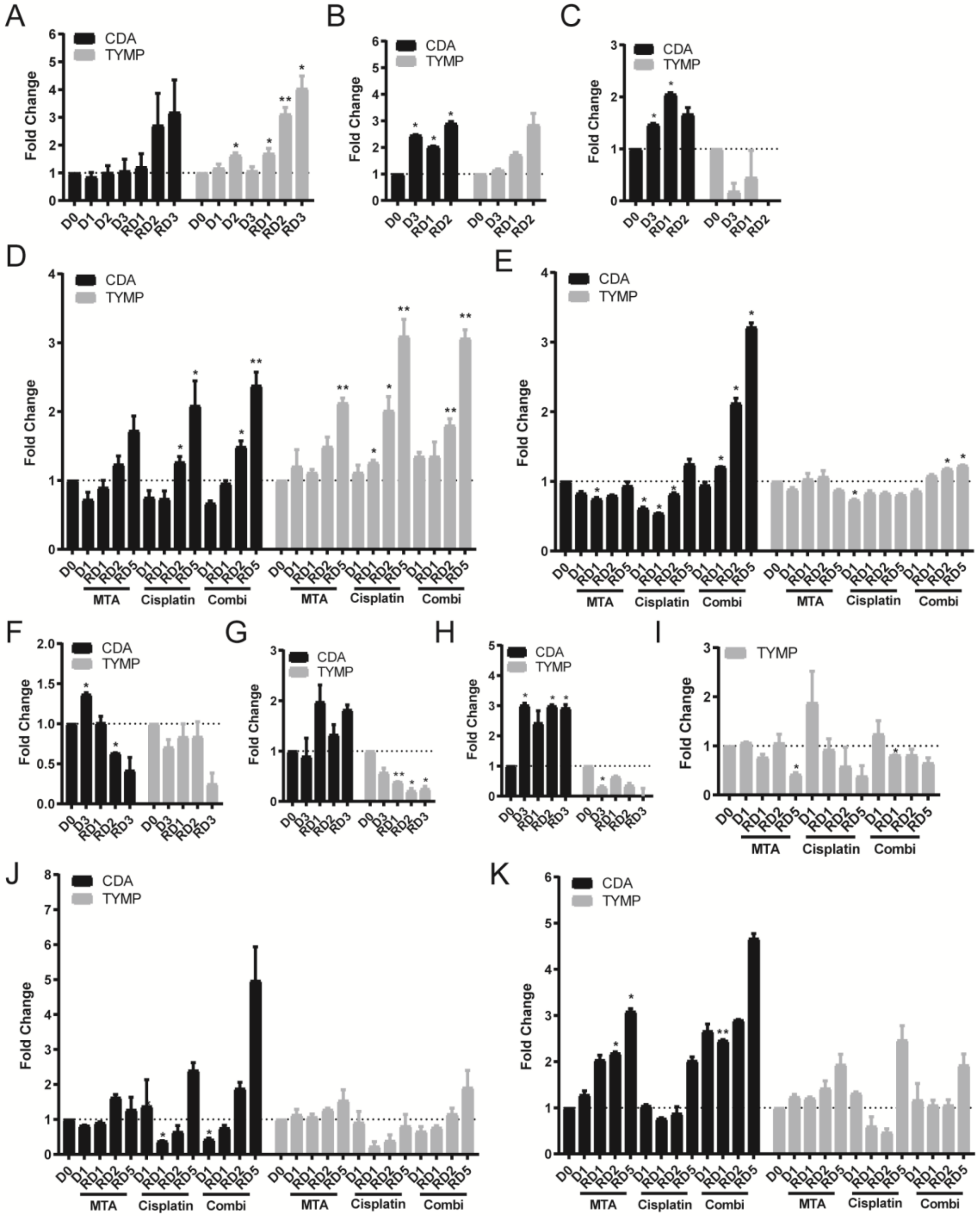

Supplementary Figure 7

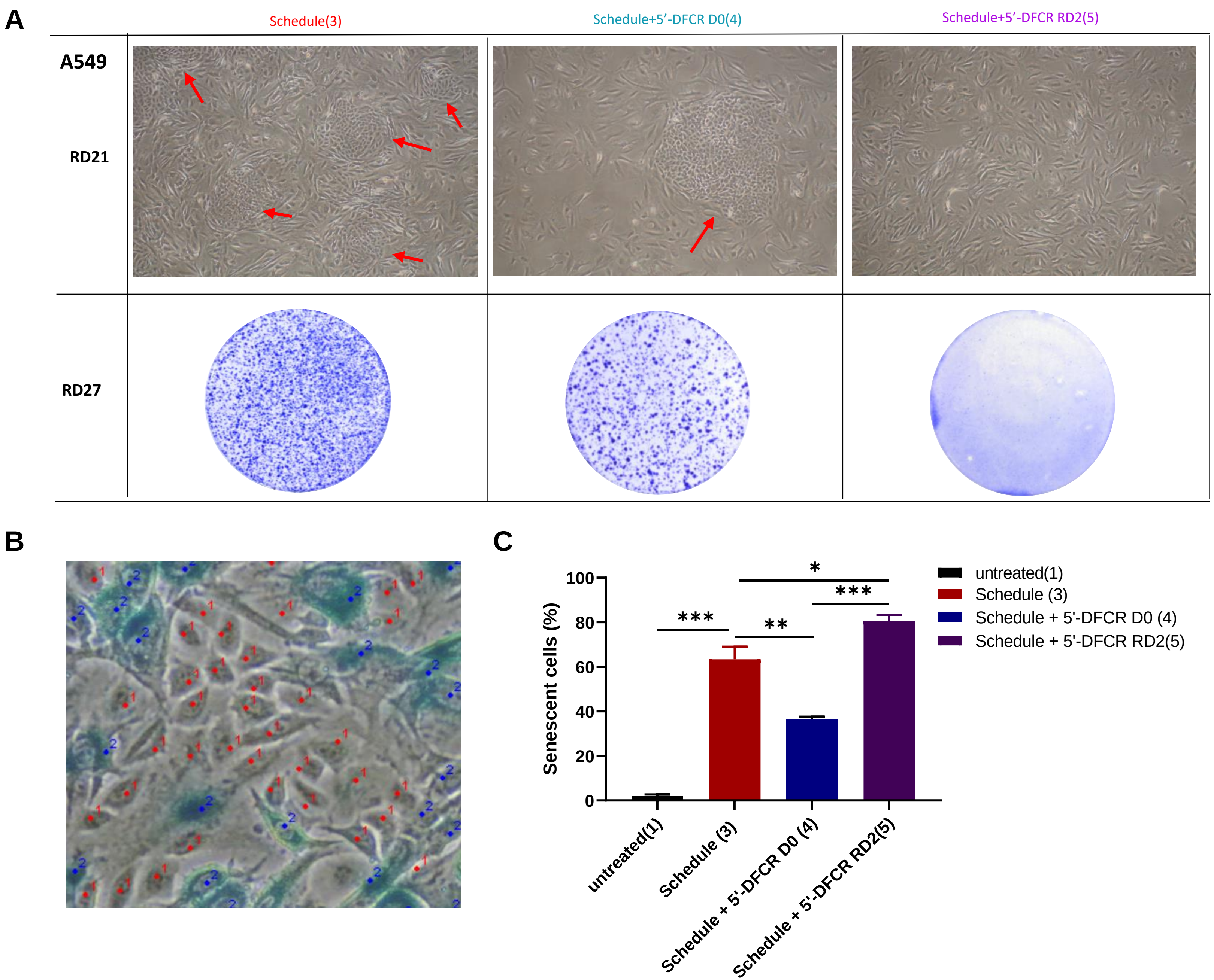

Supplementary Figure 8

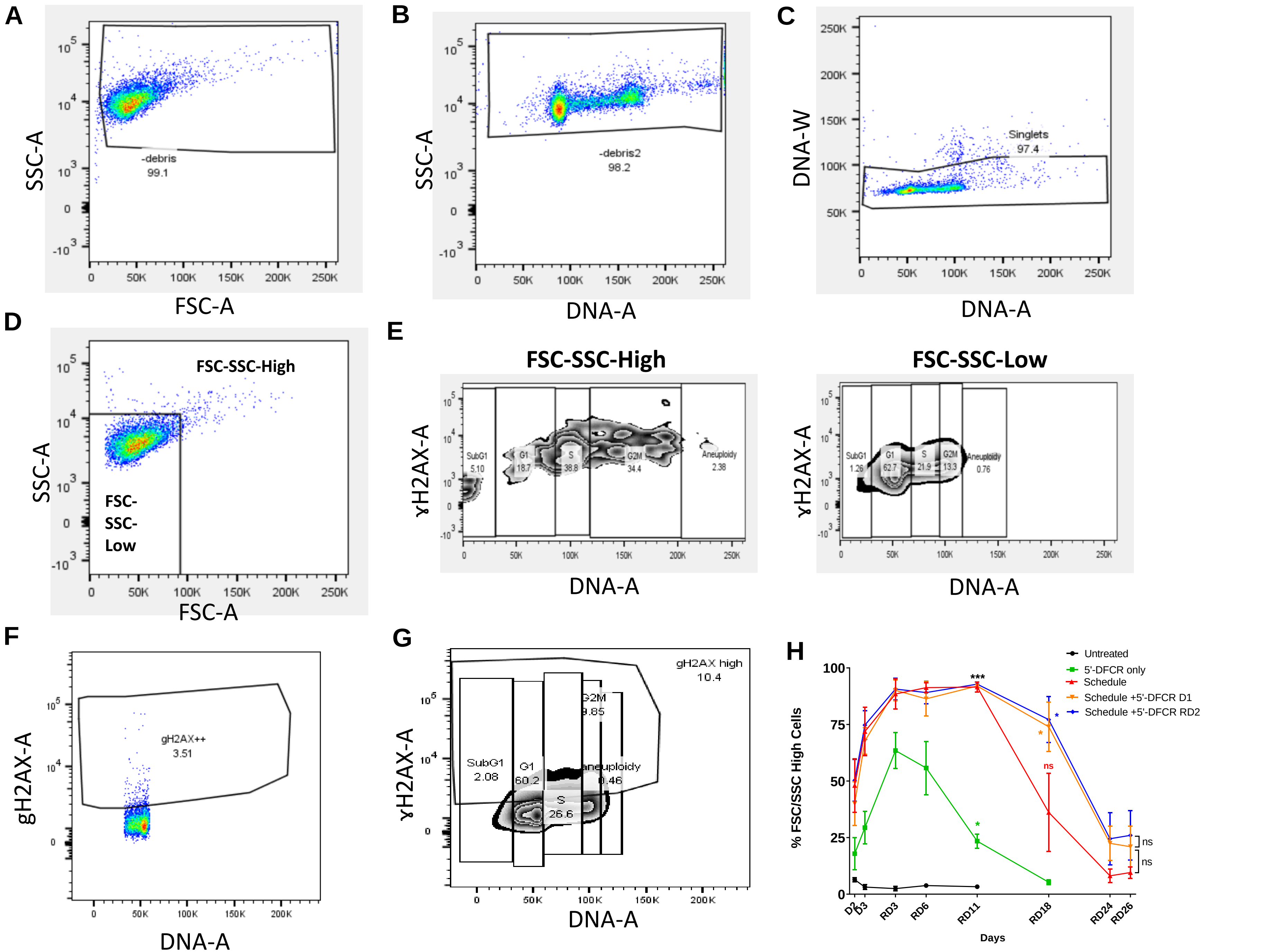

Supplementary Figure 9

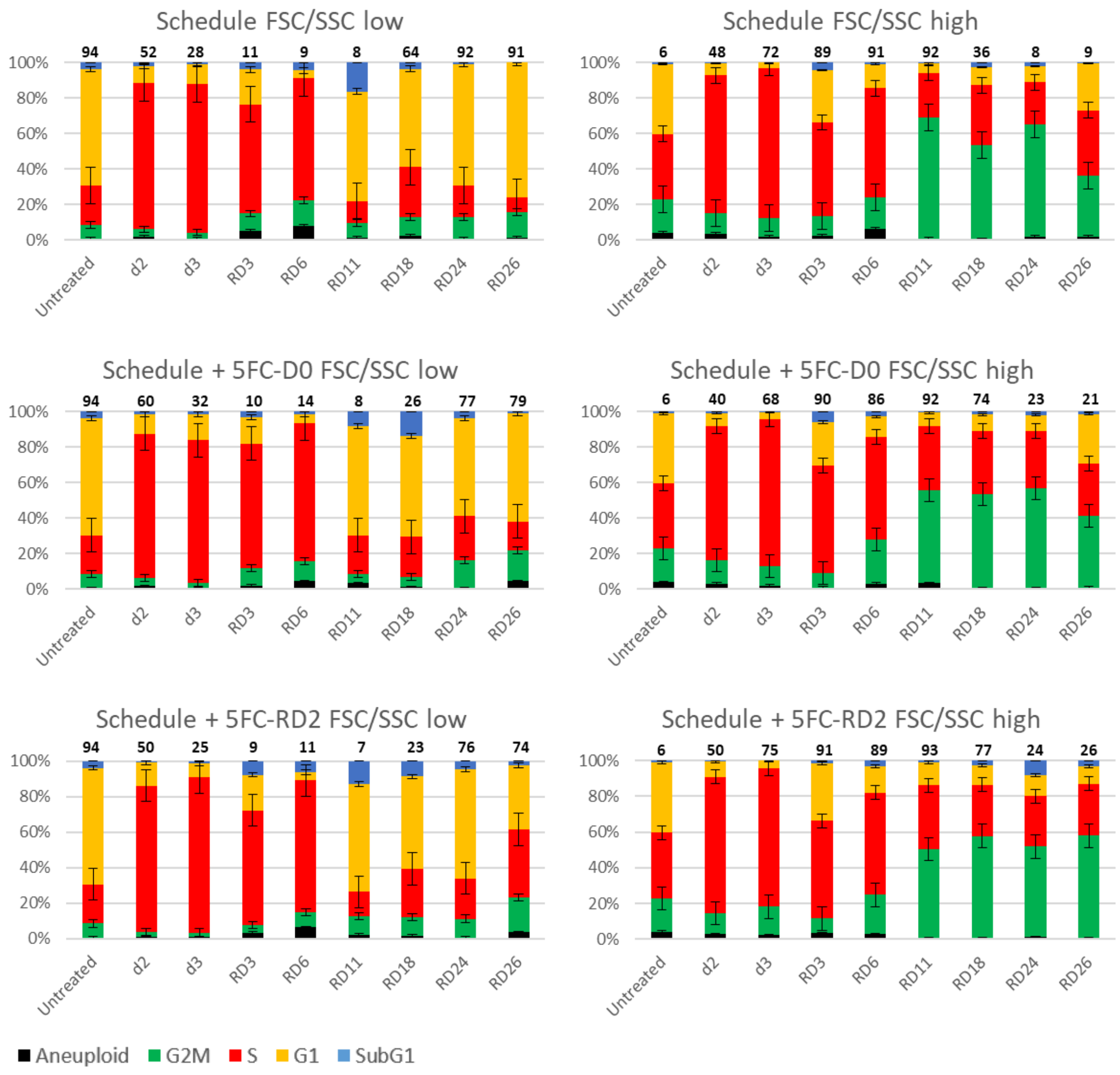

Supplementary Figure 10

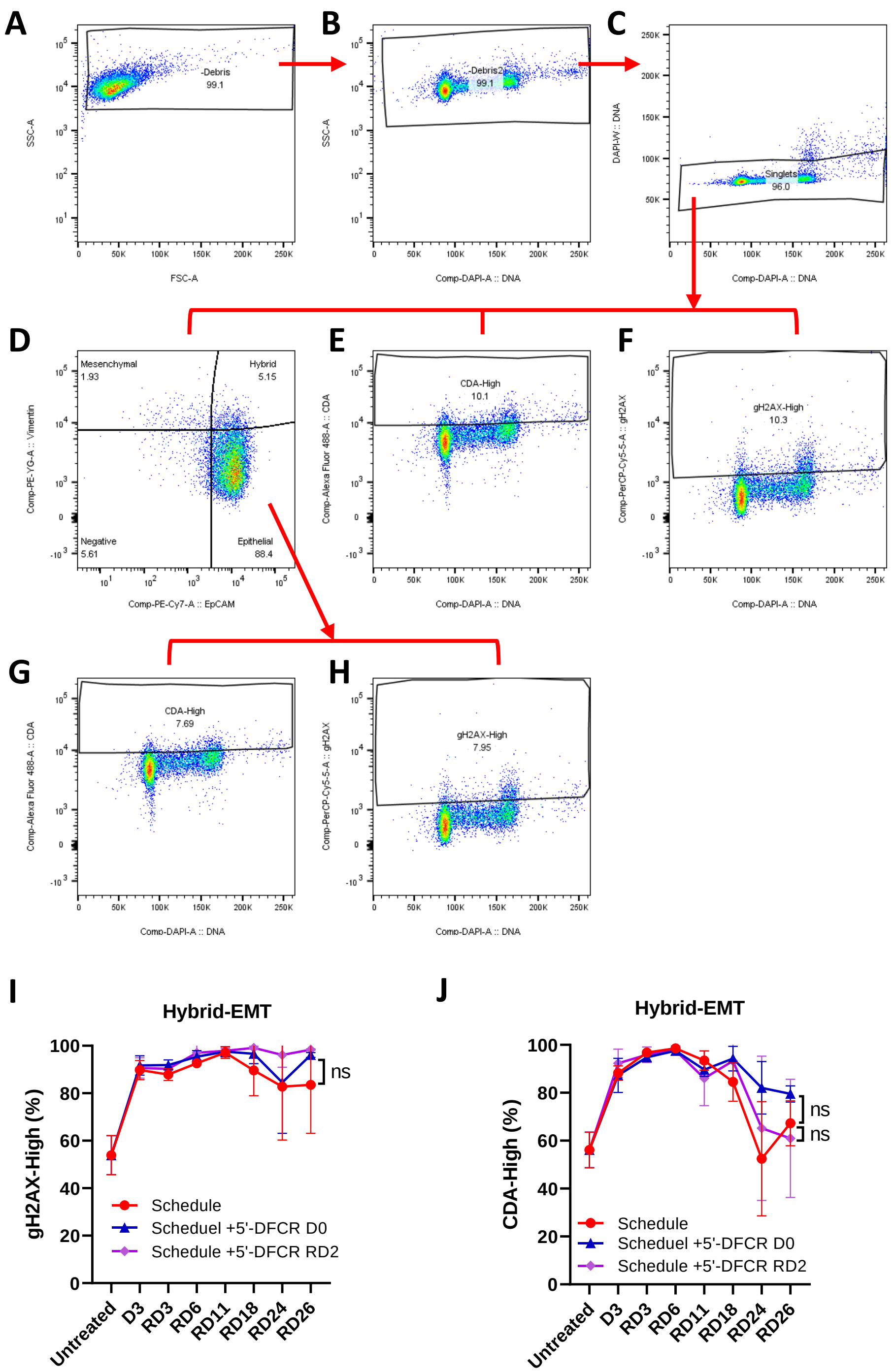

Supplementary Figure 11

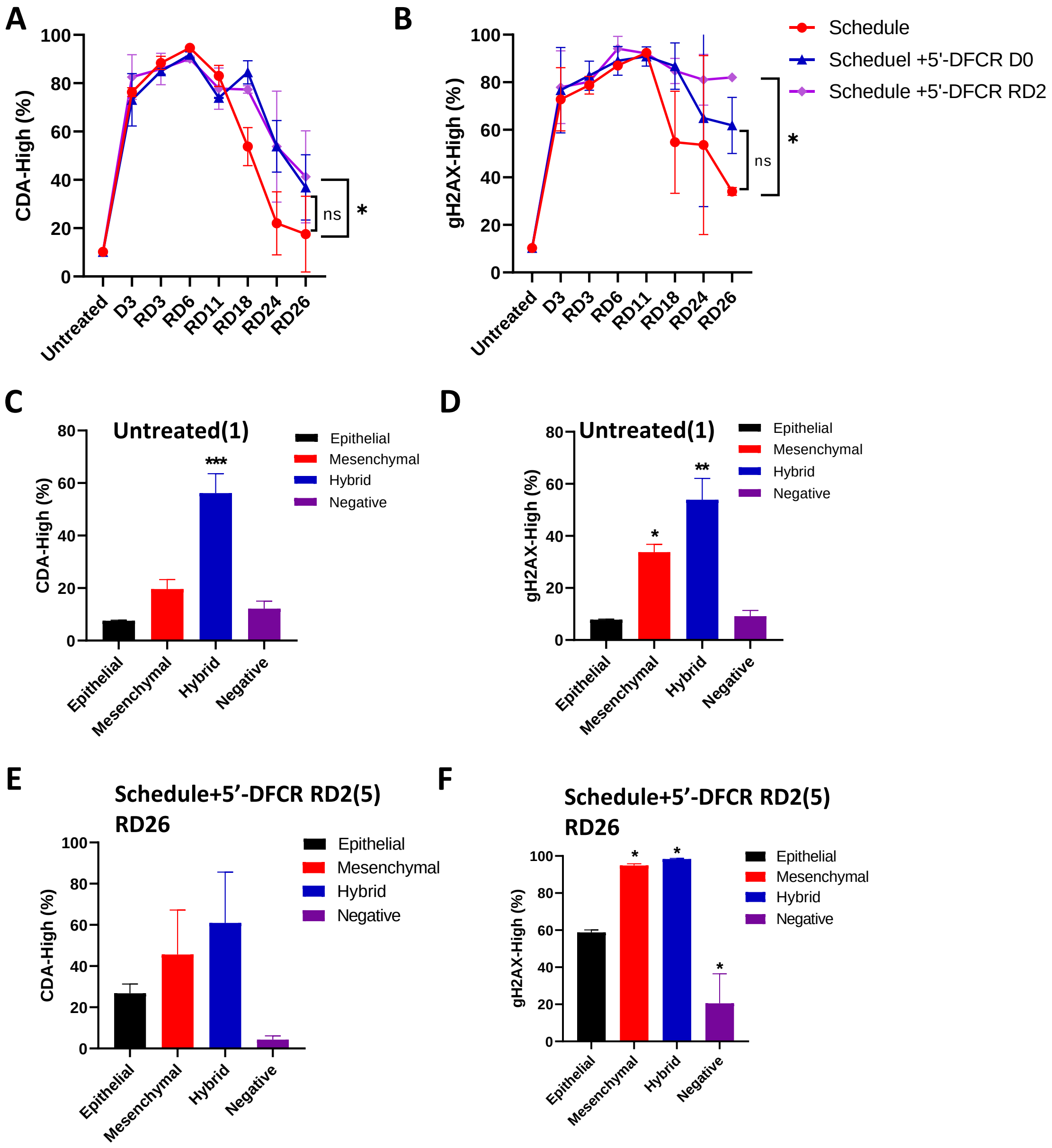

Supplementary Figure 12

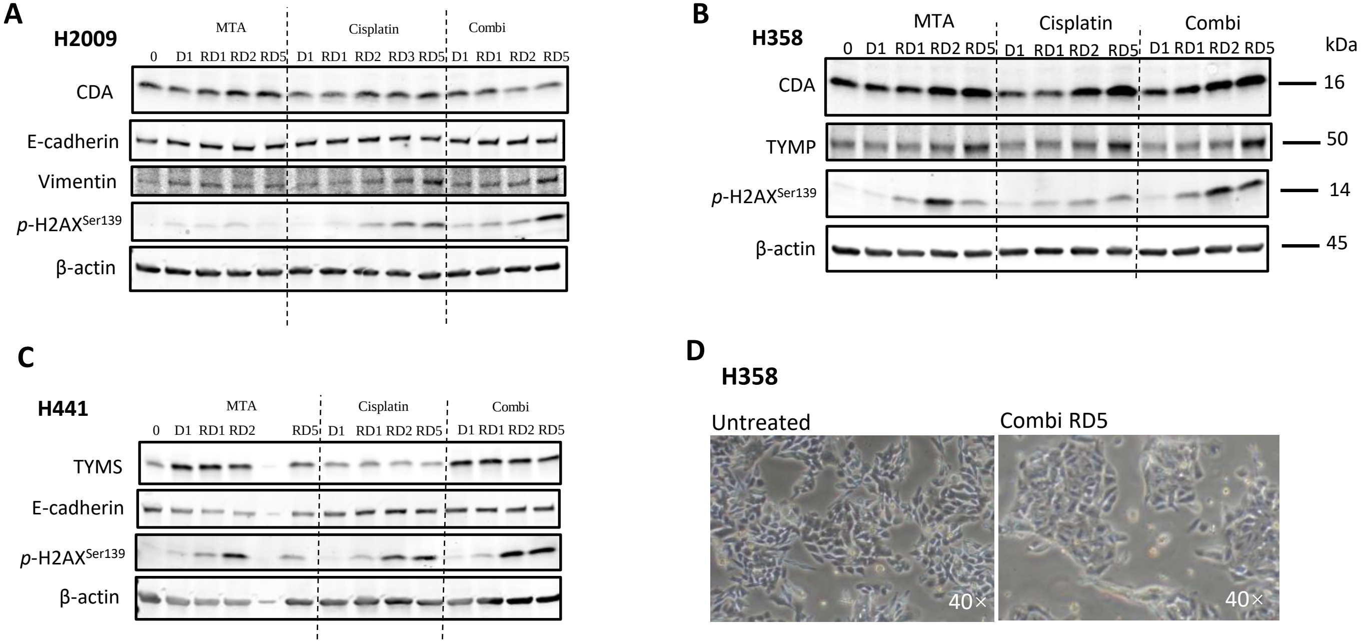

Supplementary Figure 13

A

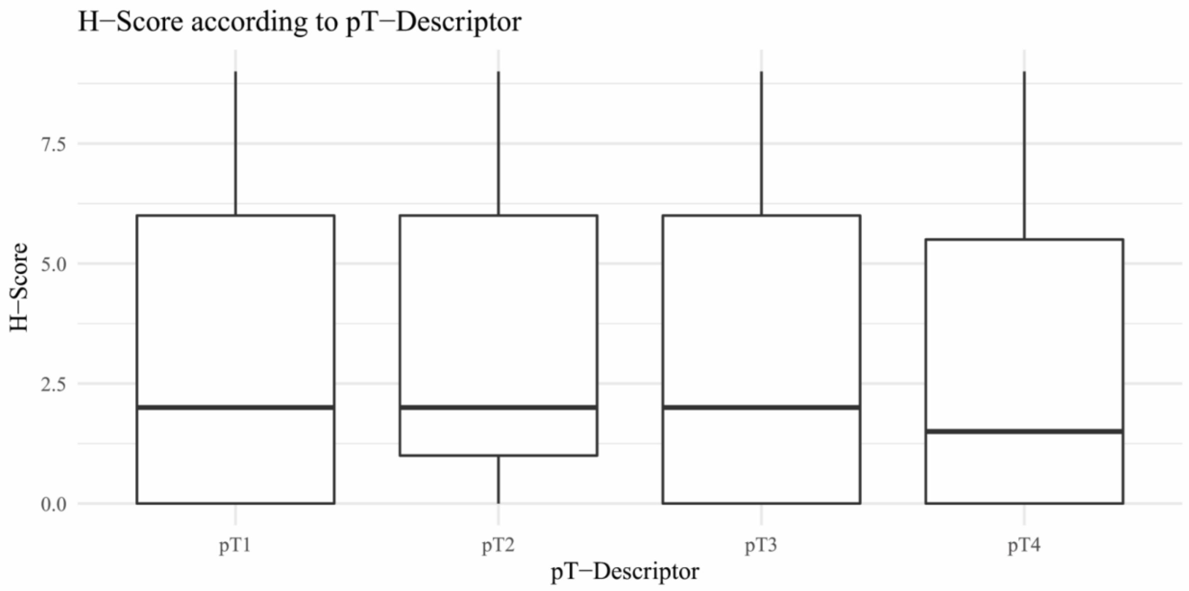

B

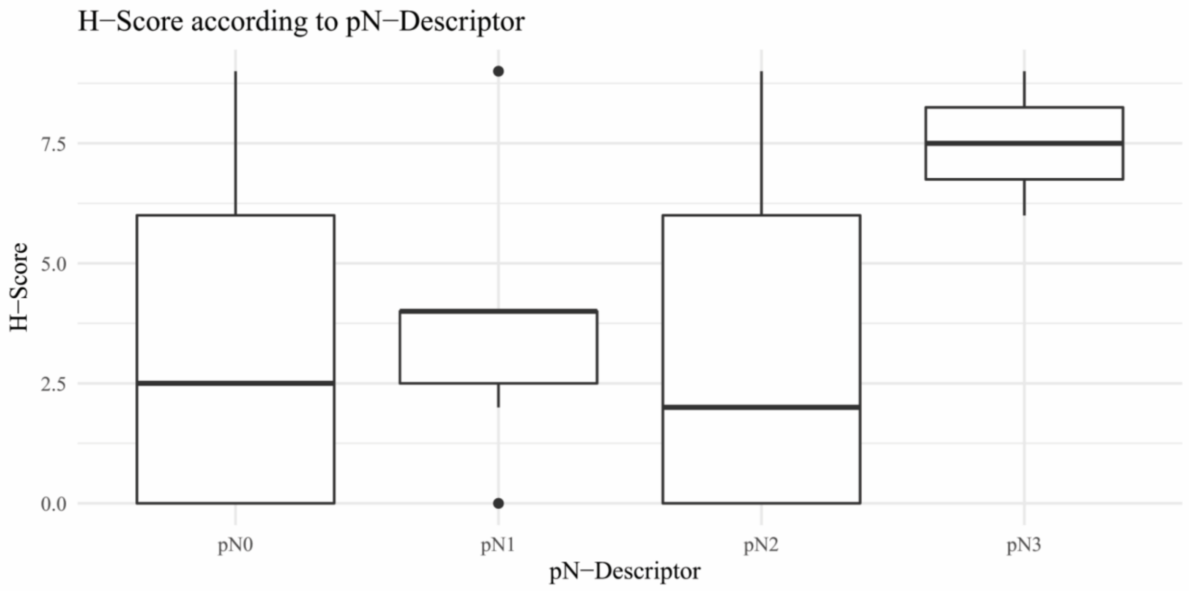

Supplementary Figure 14

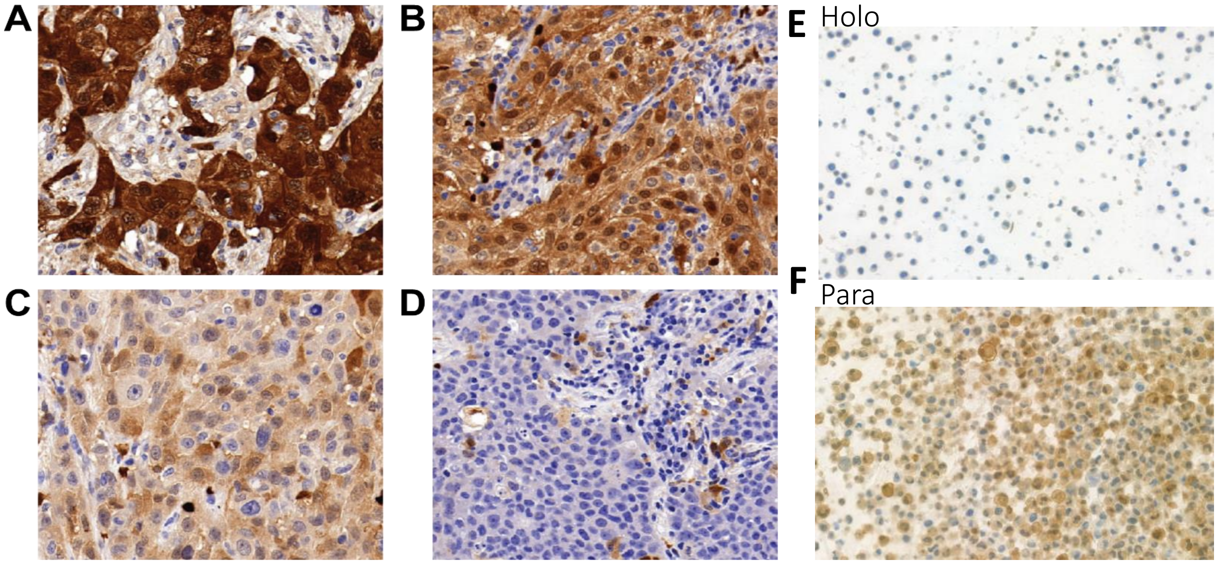

Supplementary Figure 15

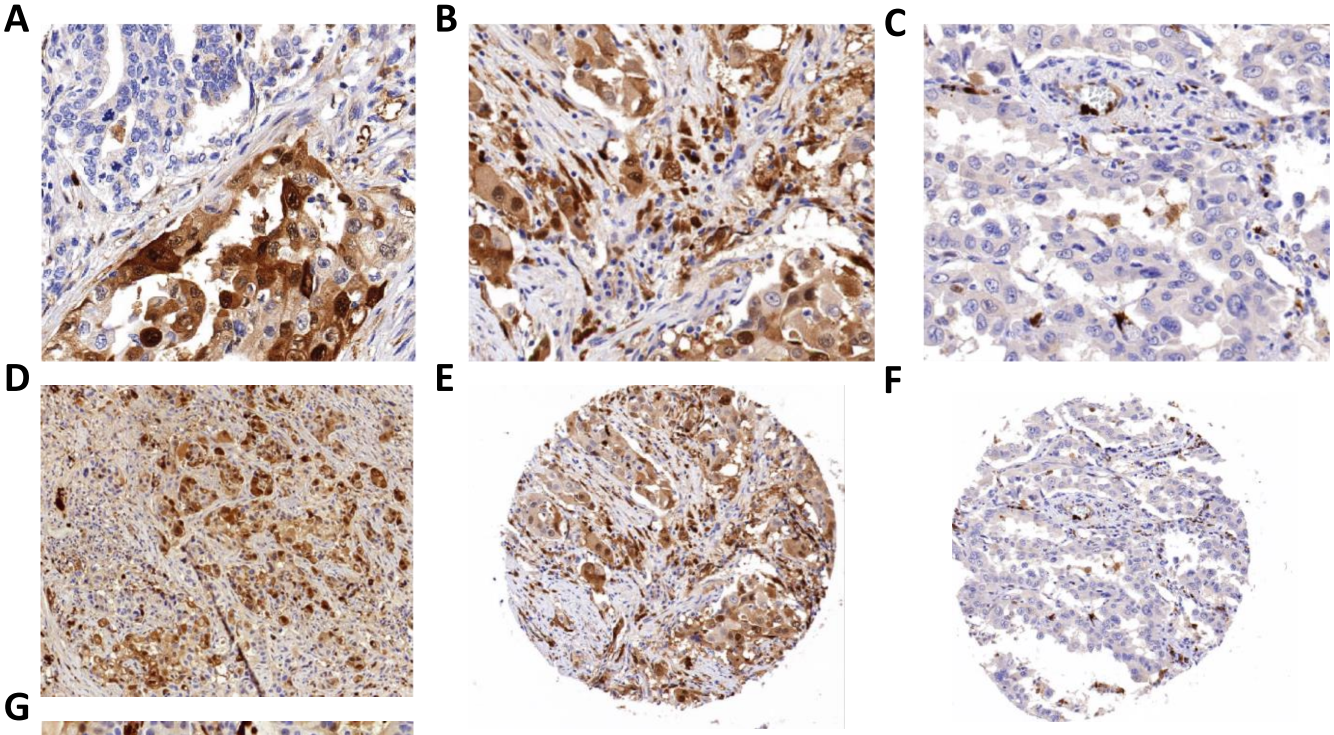

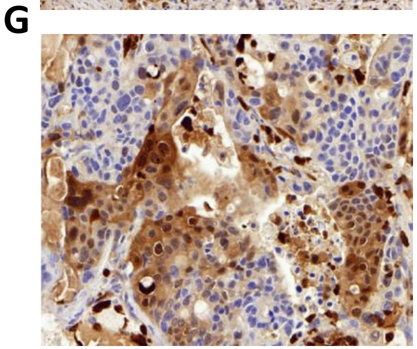

H

Staining heterogeneity depending on treatment				
		Specimen		
		Neoadjuvant	Primary	**Total**
Staining	Heterogeneous	28	42	**70**
	Homogeneous	21	18	**39**
	**Total**	**49**	**60**	**109**
