# Supplementary material for: Chemotherapy-induced CDA expression renders resistant non-small cell lung cancer cells sensitive to 5′-deoxy-5-fluorocytidine (5′-DFCR)
Source: J Exp Clin Cancer Res. 2021 Apr 19;40:138. doi: 10.1186/s13046-021-01938-2 (PMC8056724; doi:10.1186/s13046-021-01938-2)
Supplement: Supplementary file 3 — Additional file 3 : Table S1. Key resources [file 13046_2021_1938_MOESM3_ESM.pdf]

Table S1 KEY RESOURCES

REAGENT OR RESOURCE	SOURCE	IDENTIFIER	USAGE
**Antibodies**			
Anti-CDA	Atalas Antibodies	Cat. #HPA064202	0.4 μg/ mL (WB), IHC
Anti-TYMP	Atalas Antibodies	Cat. #HPA000530	0.4 μg/ mL (WB), IHC
Anti-E-cadherin	Cell Signaling Technology	Cat. #14472S	1:1000(WB)
Anti-Vimentin	Cell Signaling Technology	Cat. #5741	1:1000
Anti-TYMS	Cell Signaling Technology	Cat. #9045	1:1000
Anti-*p*-H2AX^{Ser139}	BioLegend	Cat. #613402	1:1000
Anti-CD90 PE-CF594	BD Bioscience	Cat. #562385	2.5 μL/200 μL
Anti-Sox2 AF-488	Invitrogen	Cat. #53981182	1 μL/200 μL
Anti-EpCAM PE-Cyanine 7	eBiosciences	Cat. #25-9326-42	2.5 μL/200 μL
AF546-conjugated Anti-Vimentin	Santa Cruz Biotechnology	Cat. #sc-6260 AF546	2 μL/200 μL
AF-488-conjugated Anti-Rabbit	Invitrogen	Cat. #A11034	1 μL/200 μL
PerCP-eFluor 710-conjugated Anti-*p*-H2AX ^{Ser139}	Thermo Fisher Scientific	Cat. #46-9865-42	2.5 μL/ 200 μL
Anti-β-actin	Cell Signaling Technology	Cat. #3700S	1:5000
IRDye 800CW-conjugated goat anti-rabblit IgG	Li-COR Biosciences	Cat. #926-32211	1:5000
IRDye 680LT-conjugated goat anti-mouse IgG	Li-COR Biosciences	Cat. #926-68020	1:5000
**Drugs and Inhibitors Used**			
Pemetrexed (MTA)	Elli Lilly	ALIMTA	-
Cisplatin	Sandoz	Cat. #44033792	-
IACS-010759	ProbeChem	Cat. #PC-35205	-
CPI-613	Sigma-Aldrich	Cat. #SML0404	-
Tigecycline	Sigma-Aldrich	Cat. #PZ0021	-
Tetrahydrouridine(THU)	CaymanChem	Cat. #18771-50-1	-
Teriflunomide	Selleckchem	Cat. #S4169	-
5'-deoxy-5-fluorocytidine (5'-DFCR)	CaymanChem	Cat. #10010682	-
TGF-β1	PeproTech	Cat. #AF-100-21C	5 ng/ μL or 2.5 ng/ μL
SB431542	Selleckchem	Cat. #S1067	10 μM
**Materials for Cell culture**			
Dulbecco's modified Eagle's medium nutrient mixture F- 12 Ham	Sigma-Aldrich	Cat. #D6421	-
Dulbecco's modified Eagle's medium nutrient mixture F- 12 Ham High glucose	Sigma-Aldrich	Cat. #D6429	-
RPMI-1640 Medium	Sigma-Aldrich	Cat. #R8758	
fetal bovine serum	Life Technologies	Cat. #10270-106	9 % for cell culture
Penicillin/Streptomycin solution	Sigma- Aldrich	Cat. #P0781	1 % for cell culture

L-Glutamine	Sigma- Aldrich	Cat. #25030-024	1 % for cell culture
Thermo Scientific™ Nunc™ Lab-Tek™ II Chamber Slide™ System	Thermo Scientific Nunc	Cat. NNU♯154526	-
UltraPure™ Ethidium Bromide	Thermo Fisher Scientific	Cat. #15585011	50 ng/mL
Sodium pyruvate	Sigma- Aldrich	Cat. #113246	1 mM
Uridine	Sigma- Aldrich	Cat. #58968	50 µg/mL
phosphate-buffered saline (PBS)	Insel Hospital	Cat. #1195	-
10× TrypLE	Life Technologies, ThermoFisher	Cat. #A1217702	1×
150 mm × 20 mm dishes	Bioswisstec	Cat. #20151	-
6-well plate	Falcon	Cat. #353046	-
**Materials for Western Blot**			
RIPA buffer	Cell Signaling Technology	Cat. #9806	50 µL/ well, 6-well plate
100× protease and phosphatase inhibitor cocktail	Thermo Fisher Scientific	Cat. #78440	1×
Pierce™ BCA Protein Assay Kit	Thermo Fisher Scientific	Cat. #23227	-
SDS-PAGE gel	Bio-Rad	Cat. #4561095	-
10x Tris/Glycine/SDS	Bio-Rad	Cat. #1610732	1×, for SDS-PAGE
nitrocellulose membranes	Bio-Rad	Cat. #170-4158	-
RIPA buffer	Cell Signaling Technology	Cat. #9806	50 µL/ well, 6-well plate
100× protease and phosphatase inhibitor cocktail	Thermo Fisher Scientific	Cat. #78440	1×
Intercept® (TBS) Blocking Buffer	Li-COR Biosciences	Cat. #927–60001	-
Tris Buffered Saline (TBS) BioUltra, tablet	Sigma- Aldrich	Cat. #94158-10TAB	Solution + 0.2% Tween-20
**Other Materials**			
2.3 % crystal violet solution	Sigma- Aldrich	Cat. #HT901-8FOZ	1% in 50% ethanol
PNPP Substrate	Thermo Fisher Scientific	Cat. #34045	1 mg/ mL for APH assay
Senescence β-galactosidase staining kit	Cell Signaling Technology	Cat. #9860	-
PNPP Substrate	Thermo Fisher Scientific	Cat. #34045	1 mg/ mL for APH assay
MitoTracker® Deep Red FM	Invitrogen	Cat. #M22426	200 nM (IF), 25 nM (FACS)
IC fixation buffer solution	Thermo Fisher Scientific	Cat. #00-8222-49	
FIX/PERM solution	BD Bioscience	Cat. #554722	0.5 mL/ 1×10^6 cells
Fc Receptor Binding Inhibitor Functional Grade Monoclonal Antibody	Thermo Fisher Scientific	Cat. #14-9161-73	0.25% for blocking in FC

4'6-diamidino-2-phenylindole (DAPI)	Sigma-Aldrich	Cat. #D9542-10MG	0.5 ug/ml
Prolong Gold antifade reagent	Life technology	Cat. #P36931	-
Cytidine Deaminase Activity Assay Kit (Fluorometric)	BioVision	Cat. #K451-100	-
Dounce Tissue Homogenizer	BioVision	Cat. #1998-1	-
CDA (Human) - 3 unique 27 mer siRNA duplexes - 2 nmol each	OriGene Technologies	Cat. #SR300700	-
Lipofectamine 2000 transfection reagent	Thermo Fisher Scientific	Cat. #11668027	8 uL /well, 6-well plate
GenElute™ Mammalian Genomic DNA Miniprep Kit	Sigma- Aldrich	Cat. #G1N350	-
GoTaq® qPCR Master Mix	Promega	Cat. #A6002	-
Eva Green	Biotium	Cat. #31000	1:40
CXR reference dye	Promega	Cat. #C541A	1:100
GoTaq® Long PCR Master Mix (2×)	Promega	Cat. #M4021	-
**Devices**			
microscope (Eclipse TS100)	Nikon Instruments Inc., Melville NA, USA		-
Flow cytometer	BD Bioscience	LSR2 upgraded	-
**Service**			
DNA fingerprinting	Microsynth	-	-
Image Studio Lite System	Li-COR Biosciences	-	-
